# Synergistic health effects of air pollution, temperature, and pollen exposure: a systematic review of epidemiological evidence

**DOI:** 10.1186/s12940-020-00681-z

**Published:** 2020-12-07

**Authors:** Susan C. Anenberg, Shannon Haines, Elizabeth Wang, Nicholas Nassikas, Patrick L. Kinney

**Affiliations:** 1 Milken Institute School of Public Health, George Washington University, 950 New Hampshire Ave NW, Washington, DC 20052 USA; 2 Now at: American Lung Association, Springfield, IL USA; 3 Department of Pulmonary, Critical Care, and Sleep Medicine, Brown University Alpert Medical School, Providence, RI 02903 USA; 4 School of Public Health, Boston University, Boston, MA USA

**Keywords:** Air pollution, Temperature, Pollen, Systematic review

## Abstract

**Background:**

Exposure to heat, air pollution, and pollen are associated with health outcomes, including cardiovascular and respiratory disease. Studies assessing the health impacts of climate change have considered increased exposure to these risk factors separately, though they may be increasing simultaneously for some populations and may act synergistically on health.

Our objective is to systematically review epidemiological evidence for interactive effects of multiple exposures to heat, air pollution, and pollen on human health.

**Methods:**

We systematically searched electronic literature databases (last search, April 29, 2019) for studies reporting quantitative measurements of associations between at least two of the exposures and mortality from any cause and cardiovascular and respiratory morbidity and mortality specifically. Following the Navigation Guide systematic review methodology, we evaluated the risk of bias of individual studies and the overall quality and strength of evidence.

**Results:**

We found 56 studies that met the inclusion criteria. Of these, six measured air pollution, heat, and pollen; 39 measured air pollution and heat; 10 measured air pollution and pollen; and one measured heat and pollen. Nearly all studies were at risk of bias from exposure assessment error. However, consistent exposure-response across studies led us to conclude that there is overall moderate quality and sufficient evidence for synergistic effects of heat and air pollution. We concluded that there is overall low quality and limited evidence for synergistic effects from simultaneous exposure to (1) air pollution, pollen, and heat; and (2) air pollution and pollen. With only one study, we were unable to assess the evidence for synergistic effects of heat and pollen.

**Conclusions:**

If synergistic effects between heat and air pollution are confirmed with additional research, the health impacts from climate change-driven increases in air pollution and heat exposure may be larger than previously estimated in studies that consider these risk factors individually.

**Supplementary Information:**

The online version contains supplementary material available at 10.1186/s12940-020-00681-z.

## Background

Climate change is expected to increase exposure to environmental health risk factors, including extreme temperatures, air pollution, and aeroallergens [1–5]. These environmental health risk factors are associated with a range of health outcomes, including cardiovascular and respiratory disease [5]. Changes in these risk factors will be spatially heterogeneous, depending on local emission sources, meteorology, vegetation type and distribution, and other factors. As these risk factors do not exist in isolation, populations may experience simultaneous increases in exposure to heat, air pollutants, and pollen. Understanding whether these environmental health risk factors have synergistic effects on health outcomes can inform future climate change health risk assessments. The objective of this paper is therefore to determine whether the current state of the epidemiological evidence supports the presence of synergistic effects between heat, air pollutants, and pollen on human health outcomes.

Both average and extreme temperatures are expected to increase with climate change [5]. These changes may compromise the body’s ability to regulate temperature leading to a range of health outcomes, including heat exhaustion, heatstroke, and hyperthermia [6]. Exposure to extreme heat events can worsen cardiovascular and respiratory diseases, as well as other chronic conditions, such as cerebrovascular disease, diabetes, and kidney disease [7, 8]. The mechanisms by which heat exacerbates respiratory disease are not well understood. In respiratory diseases such as asthma and chronic obstructive lung disease, inflammation plays a central role in the pathogenesis and exacerbation of the disease. Heat increases systemic and pulmonary inflammation as a consequence of thermoregulation – the attempt by the body to maintain a temperature within a safe range [9]. A second mechanism by which heat affects chronic lung disease may be related to impairment in breathing patterns meant to compensate for elevations in body temperature [10, 11]. Heat induces cardiovascular disorders through multiple mechanisms including cell damage, inflammation, and blood clotting [12]. For mortality, epidemiological studies have linked even small increases in daily mean or maximum temperatures with increases in premature death. Applying these epidemiological exposure-response relationships to climate model simulations of future temperature, studies have attributed tens of thousands of premature deaths to increasing temperatures in the United States by mid-century [13]. The most vulnerable population subgroups to heat include older adults, children, people working outdoors, and economically disadvantaged communities [7], as well as end stage renal disease patients [14]. While climate adaptation measures can lessen some of the health impacts, climate change-related temperature increases are expected to be an important health risk factor in the U.S. and globally in the future.

Air pollution exposures may also increase with climate change through various pathways, including increased frequency of stagnation events that prohibit atmospheric venting, enhanced photochemical production of secondary pollutants (e.g. tropospheric ozone and some components of fine particulate matter, PM_2.5_), and increasing “natural” gaseous and particulate emissions influenced by warmer and drier conditions (e.g. wildfire smoke, airborne soil dust, and ozone and PM_2.5_ formation from biogenic volatile organic compounds) [2]. As a result, simulations of future air quality under various climate change scenarios indicate a likely “climate penalty” for ozone, making it harder to attain ambient air quality standards even with the same level of anthropogenic emission controls in place [15, 16]. The literature is more mixed for the effects of climate change on PM_2.5_ given the varied and often counteracting effects of climate on PM_2.5_ components and precursor emissions, as well as atmospheric transport and loss. Recent studies suggest a potentially large influence of wildfire smoke and airborne soil dust on PM_2.5_ concentrations [17]. Air pollution exposure can have large implications for human health, particularly heart and lung disease and mortality, through various mechanisms. Exposure to air pollutants, such as PM_2.5_ and ozone, increases oxidative stress leading to pulmonary and systemic inflammation and increased permeability of the lung lining (airway epithelium), increased airway hyperresponsiveness in asthmatics, and decreases in lung function in healthy patients and patients with chronic lung disease [17, 18]. Development and worsening of cardiovascular disease in response to air pollution exposure likely occurs along pathways that include systemic inflammation, alterations in coagulation, dysfunction in the lining of blood vessels (endothelial dysfunction), and progression of atherosclerosis [19]. Following these pathways, air pollution is associated with increased respiratory and cardiovascular mortality. Given the large body of epidemiological literature providing strong evidence for associations between PM_2.5_ and premature mortality from cardiovascular disease, respiratory disease, and lung cancer, and between ozone and respiratory mortality, even small increases in pollution levels in the future can have profound influences on human health outcomes [17, 20].

Climate change is also expected to affect the start, duration, and intensity of the pollen season, with changes differing by region [21]. Climate change and rising greenhouse gas concentrations are correlated with aeroallergens in a number of ways, including increased and faster plant growth, increased pollen production by plants, increased allergenic proteins contained in pollen, earlier start time of plant growth, and longer plant seasons [22]. Meteorological conditions, including precipitation, atmospheric temperature, humidity, and wind speed, can alter the concentrations of plant pollens, which can then influence the occurrence of allergic diseases [23]. Inhalation of pollen grains causes disruption of the immune system within the lungs and increases the susceptibility of individuals to respiratory viral infections [24]. These breakdowns in immune system defenses following exposure to pollen are seen not only in patients with underlying allergies, but also in healthy individuals. In asthmatics, exposure to pollen activates an array of immune cells resulting in bronchoconstriction and increased permeability of airway epithelium [25]. There are few studies that have examined the link between aeroallergen exposure and cardiovascular disease; however, airborne pollen may be a risk factor for myocardial infarction [26]. The mechanism may be related to pollen triggering mast cell activation and histamine release leading to coronary artery spasm or plaque rupture. With the pervasiveness of allergies and allergic asthma among diverse populations throughout the U.S. and the world, climate-related changes in aeroallergen exposure may have widespread impacts on allergic rhinitis and asthma emergency department visits, both of which place a heavy burden on the U.S. healthcare system.

There is substantial literature on respiratory and cardiovascular outcomes related to the isolated exposure to heat, air pollution, or pollen [12, 27–29]. However, fewer studies examine potential synergies or mechanisms behind interactions among these environmental risk factors. There is evidence that air pollutants can bind to pollen grains, precipitating faster release of allergens, increasing allergen absorption in the lungs, and potentiating the allergenicity of pollen, however this is mostly supported in in vitro and animal studies and the clinical significance on a population level is less certain [17, 30, 31]. Prior studies suggest a joint effect of air pollution and heat on health outcomes such as mortality and respiratory morbidity [32]. Many disease states, including heart and lung disease, share a common pathway in which exposure to heat, air pollution, and pollen causes systemic and organ-specific inflammation and cellular damage [9, 17, 28, 33].

Previous studies assessing the potential health impacts of future climate change have considered heat, air pollution, and pollen exposure individually and have not accounted for potential synergistic effects [7, 34–40]. For example, the comprehensive Climate Change Impacts and Risk Analysis project for the U.S. includes estimates of future increases in heat-related mortality, ozone-related mortality, and asthma emergency department visits attributable to aeroallergens, with substantial increases simulated for moderate and severe climate scenarios [4, 41]. Each of these risk factors was considered separately when estimating future health impacts. If there are synergistic effects between these exposures, using single-hazard approaches may underestimate the health impacts of heat, air pollution, and pollen exposures under climate change.

Here, we conduct a systematic literature review of epidemiological studies to determine whether simultaneous exposure to heat, air pollution, and pollen (or a subset of these risk factors) synergistically increases the risk of mortality from any cause and mortality and morbidity of cardiovascular and respiratory disease specifically. We focus on these three risk factors as they share common attributes – they are conditions of the ambient air and have been found to affect respiratory and cardiovascular health. Other risk factors associated with climate change may also affect these health systems, but we consider the body of literature to be too nascent to support a more inclusive systematic review. Results of our review may be useful to more comprehensively characterize future public health disease burdens under climate change scenarios.

## Methods

### Search strategy, study selection, and data extraction

We conducted a systematic literature review using the Navigation Guide, a methodology for evaluating environmental evidence based on methods used in the clinical sciences [42]. The objective of this systematic review is to assess whether there are interactions between exposure to criteria air pollutants, extreme heat, and pollen, or a subset of these three risk factors, on cardiovascular or respiratory outcomes in human populations. Criteria air pollutants include ground-level ozone (O_3_), carbon monoxide (CO), nitrogen dioxide (NO_2_), lead, particulate matter (PM), and sulfur dioxide (SO_2_).

We define the “Population”, “Exposure”, “Comparator”, and “Outcomes” (PECO) statement as:

- Population: Any human population of any age in any location.
- Exposure: Areas where populations are simultaneously exposed to a) criteria air pollutants and extreme heat; b) criteria air pollutants and pollen; c) pollen and extreme heat; or d) all three risk factors.
- Comparator: Areas where these simultaneous exposures are not occurring.
- Outcome: Cardiovascular and respiratory diseases or mortality.

We searched the databases PubMed, ProQuest, and Scopus with the search terms “air pollution”, “air quality”, “air pollutants”, “pollen”, “aeroallergens”, “temperature”, “heat”, “dust”, “NO_2_”, “SO_2_”, “particulate matter”, “ozone”, “multipollutant” for exposures, and the terms “cardiovascular”, “respiratory”, “mortality”, “asthma”, and “allergies” for outcomes (Table S1). We conducted a first search on April 22, 2019 and an updated search with more search terms on April 29, 2019. We found additional articles through hand searching the references of fully screened articles.

We included original studies that measured at least two of the exposures (heat, air pollution, and pollen) and at least one of the health outcomes (cardiovascular or respiratory disease or mortality), without limiting by publication date. We excluded studies that were not published in English, did not study a human population, did not measure at least two of the exposures, did not report quantitative results for exposure-response relationships, or did not describe interactions between the exposures. We screened for reference duplicates using Mendeley Desktop. When it was not clear whether studies met the inclusion criteria or not, two reviewers discussed each study and came to a joint decision on inclusion or exclusion.

### Data extraction and risk of bias for each included study

Two authors independently extracted data and analyzed risk of bias for each included study. A third author reviewed all studies to resolve discrepancies between the two independent reviewers’ risk of bias ratings. We evaluated risk of bias for each of our included studies using the Cochrane Collaboration’s “Risk of Bias” tool and the Agency for Healthcare Research and Quality’s domains [43]. The domains we evaluated included study design, exposure assessment (air pollution), exposure assessment (temperature), exposure assessment (pollen), detection of outcome, reporting, and conflict of interest. Study design was rated as “low” risk of bias if it was a cohort, case crossover, or time series design. To be rated as “low” risk of bias for air pollution exposure assessment, the study must have measured at least two criteria pollutants and must have measured them in a way that represented individual exposure. To be rated as “low” risk of bias for pollen exposure assessment, the study had to use a method that measured pollen exposure at an individual level. To be rated as “low” risk of bias for temperature, studies had to use data from meteorological surveillance networks; we did not judge a lack of individual exposure measurement to introduce high risk of bias for temperature since temperature is less spatially heterogeneous compared with air pollution. To be rated as “low” risk of bias for detection of health outcome, the study had to use the *International Classification of Diseases* (ICD) to classify the health outcome category. To be rated as “low” risk of bias for reporting, the study had to report all outcomes that were assessed. To be rated as “low” risk of bias for conflict of interest, the study had to acknowledge that there was no conflict of interest. The possible ratings for the studies for each domain were “low”, “probably low”, “probably high”, or “high” risk of bias. We used the “probably low” and “probably high” categories when not enough information was given to definitively assign “low” and “high” ratings.

### Quality and strength of evidence across studies

To evaluate the quality and strength of evidence across all studies, we used the Grading of Recommendations Assessment, Development and Evaluation (GRADE) systematic review approach [44]. We stratified papers by the following categories of multiple exposures: 1) heat, air pollution, and pollen; 2) heat and air pollution; 3) air pollution and pollen; and 4) heat and pollen.

To evaluate the quality of the evidence across all studies, we upgraded and downgraded studies according to several criteria. Downgrading factors included serious risk of bias, serious indirectness in the studies such that evidence is not directly comparable to our PECO statement criteria, serious inconsistency in effect estimates across studies, serious imprecision due to small sample size and/or small outcome count, and likely publication bias resulting in an over or underestimate of true effects from exposure. Downgrading for serious risk of bias by − 1 occurred if there were instances of an unclear limitation in the evidence and by − 2 if there were instances of serious limitations or very serious limitations during the assessments. Downgrading for inconsistency by − 1 occurred if there were minimal or no overlap of confidence intervals and by − 2 if there was wide variance of point estimates across studies. Downgrading for indirectness by − 1 was applied if there were large differences in study population and by − 2 if there were large differences and if surrogate outcomes were applied. Downgrading for imprecision by − 1 occurred if there was a small sample size or small outcome count and by − 2 if there was both.

Upgrading factors included large magnitude of effect such that confounding alone could not explain the association, consistent dose-response gradient across studies, all plausible confounding would reduce a demonstrated effect, and all possible confounding would suggest a spurious effect when the actual results show no effect. After considering the upgrading and downgrading factors, the studies were then given a rating of “low quality”, “moderate quality”, or “high quality.” Possible ratings were 0, meaning no change from initial quality rating, − 1 or − 2, meaning downgrades in quality rating, and + 1 and + 2, meaning upgrades in quality rating. Upgrading for large magnitude of effect by + 1 occurred with the effect estimate was large such as a relative risk of 2 or higher and by + 2 if there was a very large effect estimate such as a relative risk of 5 or higher. Upgrading for dose-response by + 1 was applied if there was observation that there was a dose response gradient between increased exposure and increased outcomes and by + 2 if there was a rapid and large absolute increase in outcomes as dose increased. Upgrading for effect of plausible confounding by + 1 was applied if the plausible confounders were adjusted for in the analysis.

We evaluated the strength of evidence across all studies based on quality of the evidence, direction of effect estimates, confidence in effect estimates, and other attributes [45]. To the extent possible, we discuss these ratings according to categories of health outcomes (e.g. all-cause mortality, cardiovascular disease, and respiratory disease). The ratings for strength of the evidence are: “evidence of lack of association” (studies show no adverse effect), “inadequate evidence” (studies permit no conclusion about an effect), “limited evidence” (studies suggest an effect but only in a single or limited number of studies), and “sufficient evidence” (studies indicate a causal relationship between exposure and effect). We followed the more detailed definitions of each strength rating given by Johnson et al. [46].

## Results

**Fig. 1:**
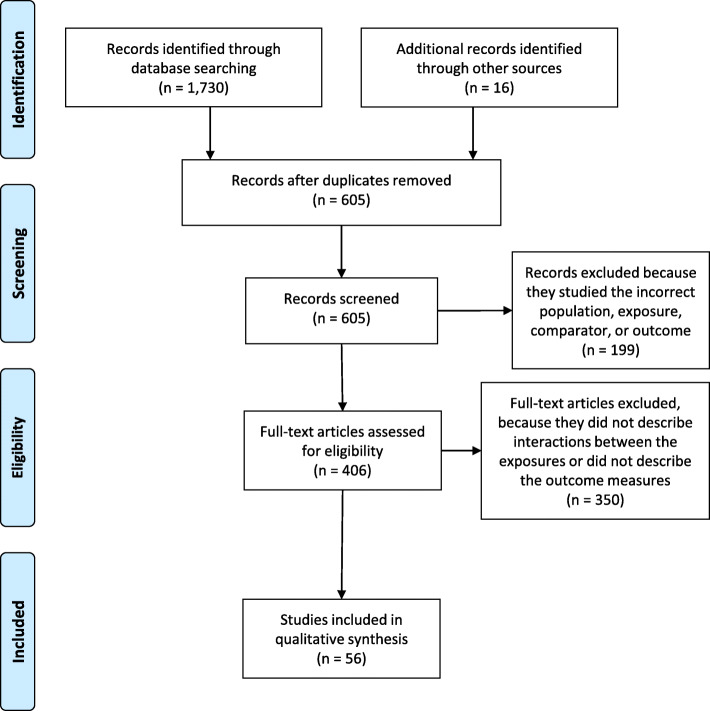
PRISMA Diagram showing the number of studies included and excluded at each step

Our search retrieved 1730 unique records, and we added 16 papers identified through other sources (Fig. 1). We screened 605 papers after removing duplicates and assessed the full text of 406 articles for eligibility. We excluded 350 articles because they did not describe interactions between the exposures or did not describe the outcome measures. Ultimately, we included 56 studies that met our eligibility criteria. Table 1 includes descriptions of each study.

**Table 1:** Descriptive information for all included studies, categorized by the combination of risk factor exposures

Study	Type	Location	Duration	Outcome	Population	Pollutants Measured	Pollen Measured	Temperature Measurement
Air pollution, heat, and pollen (*n* = 6)								
Respiratory								
Hebbern 2015 [47]	Time series	10 Canadian cities	Apr 1994- Mar 2007	Asthma hospital admissions	Not reported	CO, O_3_, NO_2_, SO_2_, PM_10_, PM_2.5_	Weed, tree, grass	Daily Mean
Makra 2015 [48]	Time series	Szeged, Hungary	1999–2007	Asthma emergency room visits	0–14 years; 15–64 years; 65+ years (*n* = 936 asthma ER visits)	CO, NO, NO_2_, SO_2_, O_3_, PM_10_	Ambrosia, maple, alder, mugwort, birch, hemp, hornbeam, goosefoot, hazel, ash, walnut, mulberry, pine, plantain, platan, grasses, poplar, oak, dock, willow, yew, linden, elm, nettle	Daily Mean, daily maximum, daily minimum, daily range
Matyasovszky 2011 [49]	Time series	Szeged, Hungary	1999–2007	Respiratory hospital admissions	All ages; 15–64 years; 65+ years (*n* = 133,464 hospital admission)	CO, NO, NO_2_, SO_2_, O_3_, PM_10_	Ambrosia, maple, alder, mugwort, birch, hemp, hornbeam, goosefoot, hazel, ash, walnut, mulberry, pine, plantain, platan, grasses, poplar, oak, dock, willow, yew, linden, elm, nettle	Daily mean, maximum, minimum, range
Mazenq 2017 [50]	Nested case control	Southeastern France	Jan 2013-Dec 2013	Asthma emergency room visits	3–18 years (*n* = 1182 asthma ER visits)	PM_10_, PM_2.5_	cypress, birch, ash, grass, urticaceae	Daily average
Mireku 2009 [51]	Retrospective time series	Detroit, MI	Jan 2004- Dec 2005	Asthma emergency room visits	1–18 years (*n* = 25,401 asthma ER visits)	PM_2.5_, PM_10_, SO_2_, O_3_	Total	Daily average
Witonsky 2019 [52]	Retrospective cohort	Bronx, NY	Jan 2001- Dec 2008	Asthma emergency room visits and hospitalizations	All ages (*n* = 42,065 asthma ER visits; *n* = 1664 asthma-related hospitalizations)	NO_x_, O_3_, SO_2_	Grass, weed, tree,	Daily average
Air pollution and temperature (*n* = 39)								
Multiple health endpoints								
Analitis 2014 [53]	Ecological time series	9 European cities	1990–2004	All natural, cardiovascular, and respiratory mortality	0–64, 65–74, 75–84, and 85+ years (n not reported)	SO_2_, PM_10_, NO_2_, O_3_, CO		3-h average
Analitis 2018 [54]	Ecological time series	9 European cities	2004–2010	All natural, cardiovascular, and respiratory mortality	All ages; 15–64, 65–74, 75+ years (n not reported)	PM_10_, O_3_, NO_2_		Daily mean
Breitner 2014 [55]	Time series	Bavaria, Germany	1990–2006	Non accidental, cardiovascular, respiratory mortality	< 85, 85+ years (*n* = 338,631 deaths)	PM_10_, O_3_		Daily mean
Cheng 2012 [56]	Time series	Shanghai, China	2001–2004	Non-accidental, cardiovascular, respiratory mortality	All ages (*n* = 173,911 deaths)	PM_10_, O_3_, SO_2_, NO_2_		Daily minimum, maximum, mean
Li 2011 [57]	Time Series	Tianjin, China	2007–2009	Cardiovascular, respiratory, cardiopulmonary, stroke and IDH, Non accidental mortality	All ages; < 65, 65+ years (*n* = 111,087 deaths)	PM_10_, SO_2_, NO_2_		Daily mean
Li 2015 [58]	Time Series	Guangzhou, China	2003–2011	Non accidental mortality, cardiovascular mortality, respiratory mortality	< 65, 65+ years (*n* = 213,737 deaths)	PM_10_		Daily mean
Lokys 2018 [59]	Time series	28 districts, Germany	2001–2011	Cardiovascular and respiratory hospital admissions	Not reported	NO_2_, SO_2_, O_3_, PM_10_		Daily mean
All-cause or non-accidental only								
Burkart 2013 [60]	Time Series	Berlin and Lisbon	1998–2010	All cause mortality	Age not reported (*n* = 698,586 deaths)	PM_10_, O_3_		Hourly mean
Chen 2018a [61]	Time series	8 European cities	1999–2013	Non accidental mortality	0–74, 75+ years (*n* = 742,526 deaths)	PM_2.5_, PM_10_, O_3_		Daily mean
Chen 2018b [62]	Time Series	8 European cities; 86 US Cities	1999–2013; 1987–2000	Non accidental mortality	All ages (n not reported)	PM_10_, NO_2_, O_3_		Daily mean
Dear 2005 [63]	Time series	12 French cities	Aug-03	All cause mortality	All ages (n not reported)	O_3_		24 h Minimum, maximum
Filleul 2006 [64]	Time series	9 French cities	Aug-03	All cause mortality	All ages (n not reported)	O_3_		Daily maximum
Jhun 2014 [65]	Time series	97 cities	1987–2000	Non accidental mortality	0–99 years (n not reported)	O_3_		Daily high
Kim 2015 [66]	Time series	7 South Korean cities	Jan 2000-Dec 2009	Daily non accidental deaths	< 65, 65+ years (*n* = 828,787 deaths)	PM_10_		Daily mean
Liu 2016 [67]	Time Series	20 US communities	1987–2000	Non accidental mortality	Not reported	O_3_		Daily mean
Meng 2012 [68]	Time series	8 Chinese cities	2001–2008	Non accidental mortality	Not reported	PM_10_		Daily mean
Moolgavkar 2003 [69]	Time Series	Cook County, IL & LA County, CA	1987–1995	Non accidental mortality	All ages; 65+ years (n not reported)	O_3_, SO_2_, NO_2_, CO, PM		Daily minimum, median, maximum
Park 2011 [70]	Time series	Seoul, South Korea	Jun 1999- Dec 2007	Non accidental mortality	All ages; 65–74, 75–84, 85+ years (*n* = 291,665 deaths)	PM_10_, NO_2_, SO_2_, CO, O_3_		Daily mean, minimum, maximum
Pattenden 2010 [71]	Time series	15 conurbations in England and Wales	1993–2003	All cause mortality	0–64, 65–74, 75–84, 85+ years (n not reported)	O_3_, PM_10_		Two day Mean
Peng 2013 [72]	Time series	23 European Cities; 12 Canadian Cities; 86 US cities	Canada 1987–1996; Europe 1990–1997; US 1987–1996	Non accidental mortality	All ages; < 75, 75+ years (n not reported)	NO_2_, SO_2_, O_3_, PM_10_		Daily mean
Rainham 2005 [73]	Time series	Toronto, Canada	1981–1999	Non Trauma mortality	Not reported	CO, NO_2_, SO_2_, O_3_, PM_2.5_		Daily mean
Scortichini 2018 [74]	Time series	25 Italian cities	2006–2010	Mortality from natural causes	35+ years (*n* = 187,743 deaths)	O_3_, PM_10_		Daily mean
Shaposhnikov 2014 [75]	Time series	Moscow, Russia	2006–2009, 2010	Non accidental mortality	All ages; < 65, 65+ years (*n* = 10,860 deaths)	O_3_, PM_10_		Daily mean
Stafoggia 2008 [76]	Case crossover	9 Italian cities	1997–2004	Mortality from natural causes	35+ years (*n* = 321,024 deaths)	PM_10_		Daily mean, apparent
Sun 2015 [77]	Time Series	Hong Kong	1999–2011	Mortality from natural causes	Age not reported (*n* = 456,317 deaths)	PM_2.5_, NO_2_, SO_2_, O_3_		Daily mean
Vanos 2015 [78]	Time series	12 Canadian cities	1981–2008	Non accidental mortality	Not reported	O_3_, NO_2_, PM_2.5_, SO_2_		Daily mean
Wilson 2014 [79]	Time Series	95 US cities	1987–2000	Mortality	Not reported	O_3_		Daily mean
Zhang 2006 [80]	Time series	Shanghai, China	Jan 2001- Dec 2004	Non accidental mortality	All ages; 0–4, 5–44, 45–64, 65+ years (*n* = 173,911 deaths)	O_3_, PM_10_, SO_2_, NO_2_		Daily mean
Respiratory only								
Ding 2017 [81]	Case crossover	Taiwan	2000–2013	COPD mortality	40–64, 65–79, 80+ years (n not reported)	PM_2.5_, O_3_, SO_2_		Daily mean, maximum, minimum
Jo 2017 [82]	Time series	Busan, South Korea	2007–2010	Hospital admissions for respiratory disease	0–15,16–64, 65+ years (n not reported)	PM_2.5_, PM_10_		Daily average, minimum, maximum, range
Kunikullaya 2017 [83]	Retrospective ecological time series	Bangalore, India	One year	Asthma-related emergency room visits and hospitalizations	> 18 years (n not reported)	SO_2_, NO_2_, PM_10_, PM_2.5_		Daily mean
Lam 2016 [84]	Time series	Hong Kong	2004–2011	Asthma hospitalizations	< 5, 5–14, 15–59, 60+ years (*n* = 56,112 asthma admission)	PM_10_, SO_2_, NO_2_, O_3_		Daily mean
Mirabelli 2016 [85]	Retrospective cross sectional	United States	2006–2010	Asthma symptoms	18+ years (*n* = 50,356 respondents)	PM_2.5_, O_3_		Average daily mean
Qiu 2018 [86]	Time series	Chengdu, China	Jan 2015- Dec 2016	COPD hospital admissions	All ages; < 60, 60–70, 70–80, 80+ years (*n* = 54,966 COPD admission)	PM_10_, PM_2.5_, NO_2_, SO_2_, CO, O_3_		Daily mean
Winquist 2014 [87]	Time Series	Atlanta, GA	16 years	Asthma emergency department visits	5–17 years (n not reported)	CO, NO_2_, SO_2_, O_3_, PM_2.5_		Daily minimum, maximum,
Cardiovascular only								
Lee 2018 [88]	Case crossover	Seoul, South Korea	2008–2014	Migraine emergency room visits	All ages; < 40, 40–64, 65+ years (*n* = 18,921 ER visits)	PM_2.5_, PM_10_, NO_2_, SO_2_, O_3_, CO		Hourly mean
Luo 2017 [89]	Time series	3 Chinese cities	2008–2011	Cardiovascular mortality	All ages; < 65, 65+ years (*n* = 290,593 deaths)	PM_10_, NO_2_, SO_2_		Daily minimum, maximum, mean
Ren 2008 [90]	Time Series	95 US cities	1987–2000	Cardiovascular mortality	< 65, 65–74, 75+ years (*n* = nearly 4 million cardiovascular deaths)	O_3_		Daily maximum
Ren 2009 [91]	Time series	95 US cities	1987–2000	Cardiovascular mortality	< 65, 65–74, 74+ years (n= > 4.3 million cardiovascular deaths)	O_3_		Daily maximum
Air pollution and pollen (*n* = 10)								
Respiratory								
Anderson 1998 [92]	Time series	London	Apr 1987- Feb 1992	Asthma emergency admissions	All ages; 0–14, 15–64, 65+ years (n not reported)	O_3_, NO_2_, Black smoke, SO_2_	Birch, Grass, Oak	Mean 24 h
Cakmak 2012 [93]	Time series	11 Canadian cities	Apr 1994-Mar 2007	Asthma hospital admissions	Not reported	CO, PM_2.5_, PM_10_, NO_2_, SO_2_	Tree, Weed	Mean 24 h
Chen 2016 [94]	Time-series case-crossover	Adelaide, South Australia	Jul 2003- Jun 2013	Asthma hospital admissions	0–17, 18+ years (*n* = 36,024admissions)	PM_2.5_, NO_2_, PM_10_	Ash tree, birch, cypress, eucalyptus, fruit tree, olive tree, pinus, plane tree, she-oak, wattle, chenopodiaceae, compositae, plantain, polygonaceae, salvation jane, grass	Daily average
Cirera 2012 [95]	Time series	Cartagena, Spain	Jan 1995- Dec 1998	COPD and asthma emergency room visits,	Age not reported (*n* = 1617 asthma and 2322 COPD ER visits)	SO_2_, NO_2_, TSP, O_3_	Poaceae, Urticaceae	Hourly mean
Galan 2003 [96]	Time series	Madrid, Spain	1995–1998	Asthma emergency department visits	Age not reported (*n* = 4827 asthma attacks)	SO_2_, PM_10_, NO_2_, O_3_, CO	*Olea europaea*, Plantago sp., Poaceae, Urticaceae	Daily mean
Gleason 2014 [97]	Time-stratified case-crossover	New Jersey	April - Sept 2004–2007	Asthma emergency department visits	3–17 years (*n* = 21,854 asthma ED visits)	O_3_, PM	Tree, grass, weed, ragweed	Daily mean
Goodman 2017 [98]	Time series	New York City	1999–2009	Asthma hospital Admissions	< 6, 6–18, 19–49, 50+ years (*n* = 295,497 asthma admission)	O_3_, PM	Tree, weed, total	Daily average, maximum, minimum
Krmpotic 2011 [99]	Time series	Zagreb, Croatia	Jan 2004- Dec 2006	Asthma hospital admissions	> 18 years (*n* = 4125 asthma admissions)	NO_2_, CO, PM_10_	Alder, Hazel, Birch, Hornbeam, Oak, Grasses, Ragweed	Daily minimum, maximum, mean
Ross 2002 [100]	Prospective Cohort	East Moline, IL	7 months	Peak Expiratory flow rates, respiratory symptoms, frequency of asthma attacks, asthma medication use	5–49 years (*n* = 59 people)	O_3_, PM, SO_2_	Grass, Ragweed, Total	Daily mean, Maximum
Cardiovascular								
Stieb 2000 [101]	Time series	Saint John, Canada	Jul 1992- Jun 1994, Jul 1994- Mar 1996	Cardiorespiratory emergency department visits	Age not reported (*n* = 19,821)	CO, H_2_S, NO_2_, O_3_, SO_2_, TRS	Ascomycetes, basidiomycetes, deuteromycetes, ferns, grass, tree, weed	Daily average
Heat and pollen (*n* = 1)								
Silverberg 2015 [102]	Cohort Study	United States	2006	Pediatric hay fever	0–17 years (*n* = 91,642)	–	Total	Monthly mean

Of these 56 studies, six measured air pollution, heat, and pollen; 39 measured air pollution and heat; 10 measured air pollution and pollen; and one measured heat and pollen. Forty-six studies were a time series design, three were cohort studies, one was a cross sectional design, one was a nested case control design, and five were a case-crossover design. Data collection in these studies ranged from 1987 to 2010 and publication date ranged from 2002 to 2018. The qualifying studies ranged widely in air pollutants and pollen types measured, metrics used for each exposure type (e.g. averaging times, time lags), and health outcomes (including asthma and hay fever symptoms, cardiovascular and respiratory emergency department visits and hospitalizations, cause-specific mortality, and all-cause mortality).

**Fig. 2:**
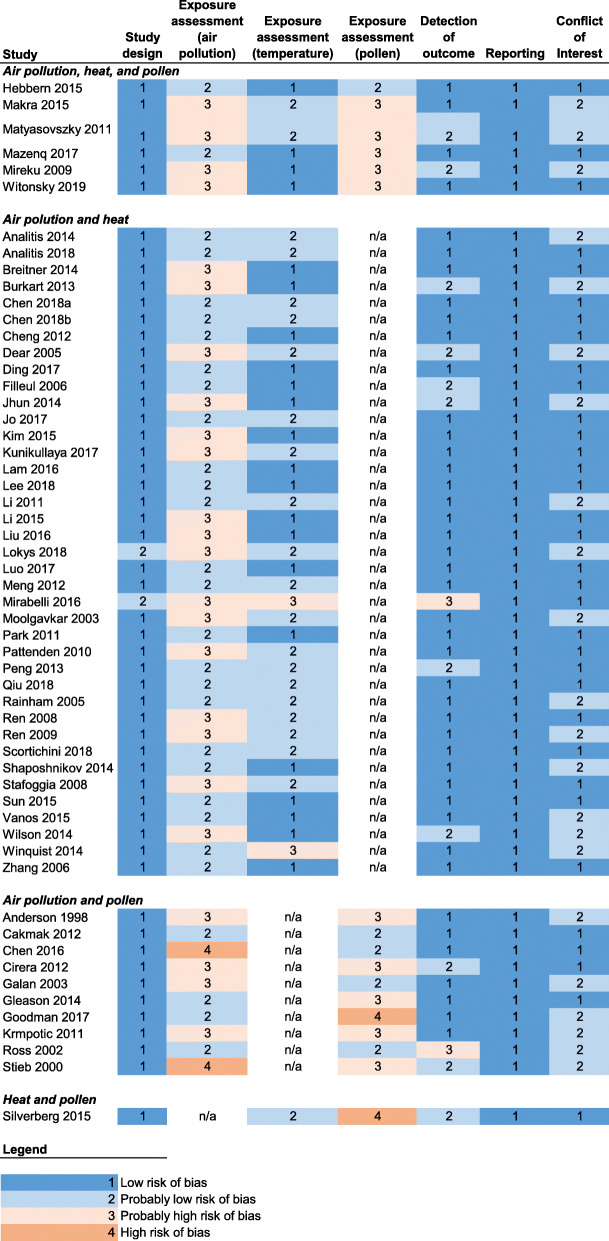
Final risk of bias evaluation for each study

Risk of bias determinations and rationale for each study can be found in Tables S2 through S57. Almost all of the studies were rated as “low” or “probably low” risk of bias for study design, detection of outcome, reporting, and conflict of interest (Fig. 2). Risk of bias for exposure assessment varied across the studies. For air pollution and pollen, we rated many studies as having a “probably high” risk due to a lack of exposure measurement at an individual level, as they used exposure assessment techniques such as central site monitors that are broadly representative of regional air pollution levels but may not represent individual exposure well. Several of these studies only used one central site monitor, which we judged could potentially introduce bias since pollution levels vary spatially within geographic areas such as cities. For temperature, studies were generally rated as having a “low” or “probably low” risk of bias since data were sourced from meteorological monitoring networks and temperature is less spatially heterogeneous compared with air pollution.

We next assessed the quality and strength of the evidence across the studies. We found six studies that examined potential interactive effects between simultaneous exposure to all three risk factors: air pollutants, pollen, and heat (Table 1). The studies were conducted in Canada, France, Hungary, and the U.S. and all focused on respiratory hospitalizations and emergency department visits (all except one focused specifically on asthma). The studies used widely different methods for categorizing temperature exposure, including spatial synoptic classification [47, 48], seasonal analysis [52], and interday temperature change [51]. Generally, the studies were individually rated as low risk of bias for most categories, including study design, detection of outcome, reporting, and conflict of interest. However, we judged some to be at probably high risk of bias for exposure assessment for both air pollutants and pollen. The findings across the studies were inconsistent, with some studies reporting interactive effects of all three or some combination of the exposures [47–49, 52], while others reported independent effects that were unaffected by controlling for the other risk factors [51] or were inconclusive when considering simultaneous exposure to all three risk factors [50].

Overall, we rated the quality of the evidence for synergistic respiratory effects between air pollution, heat, and pollen as “low” since studies were inconsistent in finding significant evidence of interactive effects and studies that reported positive associations of interactions had minimal magnitudes (Table 2). We rated the overall strength of the evidence as “limited” since synergistic effects between heat, air pollution, and pollen were observed in some studies, but these findings were not consistent across studies.

**Table 2:** Rating of the quality and strength of the evidence for studies assessing interactive effects between heat, air pollution, and pollen (*n* = 6)

**Category**	**Summary of Criteria**	**Downgrades**	**Rationale**
Initial Rating of Human Evidence = “Moderate”			
Risk of Bias	Study limitations- a substantial risk of bias across body of evidence.	-1	Downgraded because of “probably high” risk of bias for air pollution exposure assessment for four studies and for pollen exposure assessment for five studies.
Indirectness	Evidence was not directly comparable to the chosen population, exposure, comparator, and outcome.	0	Measured outcomes were assessed for humans in populations for the duration of study periods, as outlined in the PECO statement.
Inconsistency	Wide variability in estimates of effect in similar populations.	0	Some evidence of consistent effects, but the studies were too varied in definitions of risk factors and methods to judge consistency in effect estimates.
Imprecision	Studies had a small sample size and small outcome count.	0	The studies had large sample sizes with adequate samples for outcomes during study periods.
Publication Bias	Studies missing for body of evidence, resulting in an over or underestimate of true effects from exposure.	0	The studies were large studies that varied in year, data sources, and methods of statistical analysis that appeared to report outcomes found regardless of results.
**Category**	**Summary of Criteria**	**Upgrades**	**Rationale**
Large magnitude of effects	Study found confounding alone unlikely to explain association with large effect estimate as judged by reviewers.	0	Studies that reported positive associations of interactions reported effect estimates with low magnitudes.
Dose-response	Consistent relationship between dose and response in one or multiple studies, and/or exposure response across studies.	0	Studies did not report a consistent relationship between dose and response.
Confounding minimizes effect	Upgraded if consideration of all plausible residual confounders or biases would underestimate the effect or suggest a spurious effect when results show no effect.	0	No evidence that residual confounders or biases would underestimate the effect or suggest a spurious effect when results show no effect.
Overall Quality of Evidence		Low	The overall quality of the evidence supporting interactive effects is low.
Overall Strength of Evidence		Limited	An association was sometimes observed for synergy between heat, air pollution, and pollen, but the potentially high risk of bias for air pollution exposure could have impacted results and there is a lack of consistently significant findings.

We found 39 articles that examined potential interactive effects between exposure to air pollutants and heat (Table 1). These studies were carried out in Europe, the U.S., Canada, Russia, Taiwan, South Korea, India, Hong Kong, and China. Most were conducted in urban areas. A majority of the studies (29) included health endpoints that were not disease-specific, such as all-cause and non-accidental mortality. A smaller subset of 12 studies considered respiratory disease specifically (some focusing on asthma specifically) and 11 considered cardiovascular disease specifically (we have included migraine in this category as a potential indicator of cardiovascular disease, Adelborg et al. [103]). Most studies included multiple criteria pollutants – most often ozone and PM_10_, though some only included ozone, and some also included PM_2.5_, PM_2.5–10_, NO_2_, SO_2_, and CO. The temperature metric differed between studies and included daily mean, minimum and/or maximum.

Of these 39 studies addressing synergistic effects between air pollution and heat, 19 reported interactive effects between heat and air pollution exposure on health outcomes studied. Out of these studies, 15 of 29 studies examined health outcomes that were not disease-specific (e.g. all-cause mortality, hospital admissions) and found synergistic effects [53–55, 57, 58, 60, 61, 66, 68, 71, 73–77], four of 12 studies found synergistic effects for respiratory health outcomes [55, 57, 59, 84], and eight of 11 studies found synergistic effects for cardiovascular health outcomes [54, 55, 57–59, 88, 90, 91]. Here, we are not distinguishing between mortality and morbidity for respiratory and cardiovascular health outcomes. Generally, the studies found synergistic effects from simultaneous exposure to extremely high temperatures and air pollution, with a potentially additional role of relative humidity. A method of weather classification that incorporated humidity used in some of the papers was spatial synoptic classification (SSC), which is described as a “semi-automated statistical approach designed to classify complex daily weather conditions into one of six distinct categories, or a transitional category” and uses values of temperature, dew point, u and v components of wind, cloud cover, and sea level pressure [47, 48, 73, 78]. A strength of this group of studies was the large datasets of pollutant levels and meteorology, including from the National, Morbidity, Mortality, and Air Pollution Study (NMMAPS) in the United States [61, 65, 67, 90, 91] and the Ultrafine Particles and Health Study Group in Europe [61, 62]. Compared with the other categories in our review, air pollution and heat studies covered the broadest geographic area and included the largest number of people in the studies.

The evidence was strongest for synergistic effects between heat and exposure to either ozone and PM_2.5_. For ozone, 11 of 29 studies reported synergistic effects with heat [53–55, 60, 61, 71, 73, 74, 84, 90, 91]. These effects were found among inter quartile temperature analysis, seasonal analysis, and heatwave analysis in the studies. Effects were found for all-cause mortality, non-accidental mortality, cardiovascular mortality, and morbidity outcomes. High levels of ozone and high temperatures tended to be reported together and the strongest effects on outcomes were found at the highest exposures. We also found evidence for synergistic effects between heat and particulate matter, with 10 of 27 studies reporting synergistic effects [53, 54, 60, 61, 66, 73–76, 88]. These effects were found among inter quartile temperature analysis, seasonal analysis, and heatwave analysis in the studies. Effects were found for all-cause mortality, non-accidental mortality, and morbidity outcomes. A potential interactive effect between heat and particulate matter is further supported by Mazenq et al. [50], who found that temperature and particulate matter were linked but pollen was not.

While most studies assessing synergistic effects between air pollution and temperature focused on heat, several examined effects of cold [55, 56, 58–62, 67, 70, 73, 77, 79, 80, 83, 84, 86–88]. Generally, stronger results were found in warmer seasons when compared to cold seasons. Zhang et al. [80] was the only study in our review that found that synergy between ozone and the cold season was stronger than for the warm season.

We upgraded the overall quality of the evidence of synergistic effects between air pollution and heat because of the relatively consistent finding of significant exposure-response relationships showing interactive effects (Table 3). The consistent findings of interactive effects between air pollutants and heat held for all three health outcome categories considered: health outcomes that were not disease-specific (e.g. all-cause mortality), respiratory disease, and cardiovascular disease, though more studies found interactive effects for non-cause-specific endpoints and for cardiovascular disease than for respiratory disease. This result may highlight the need for more studies focusing not only on respiratory disease, but also on other diseases. These factors led us to rate the overall quality of the evidence as “Moderate” and the overall strength of the evidence as “Sufficient.”

**Table 3:** Rating of the quality and strength of the evidence for studies assessing interactive effects between heat and air pollution (*n* = 39)

**Category**	**Summary of Criteria**	**Downgrades**	**Rationale**
Initial Rating of Human Evidence = “Moderate”			
Risk of Bias	Study limitations- a substantial risk of bias across body of evidence.	-1	Downgraded due to “probably high” risk of bias for air pollution exposure assessment for 16 studies.
Indirectness	Evidence was not directly comparable to the chosen population, exposure, comparator, and outcome.	0	Measured outcomes were assessed for humans in the United States for the duration of the study periods, as outlined in the PECO statement.
Inconsistency	Wide variability in estimates of effect in similar populations.	0	There was not a wide variability in estimates of effects.
Imprecision	Studies had a small sample size and small outcome count.	0	The studies had large sample sizes with adequate samples for outcomes during study periods.
Publication Bias	Studies missing for body of evidence, resulting in an over or underestimate of true effects from exposure.	0	The studies were large studies that varied in year, data sources, and methods of statistical analysis that appeared to report outcomes found regardless of results.
**Category**	**Summary of Criteria**	**Upgrades**	**Rationale**
Large magnitude of effects	Study found confounding alone unlikely to explain association with large effect estimate as judged by reviewers.	0	Studies that reported positive associations of interactions reported effect estimates with low magnitudes.
Dose-response	Consistent relationship between dose and response in one or multiple studies, and/or exposure response across studies	1	Exposure-response relationship was directionally consistent across 15 of the 34 studies in the category.
Confounding minimizes effect	Upgraded if consideration of all plausible residual confounders or biases would underestimate the effect or suggest a spurious effect when results show no effect.	0	No evidence that residual confounders or biases would underestimate the effect or suggest a spurious effect when results show no effect
Overall Quality of Evidence		Moderate	The dose response relationships described in a number of studies did not warrant an upgrade for the overall quality rating.
Overall Strength of Evidence		Sufficient	An association was generally observed for synergistic effects of heat and air pollution exposure, specifically for ozone and PM, but the potentially high risk of bias from the air pollution exposure assessment methods in several studies could have impacted results.

We found 10 studies that assessed potential interactive effects between exposure to air pollution and pollen (Table 1). These studies were conducted in Europe, Canada, Australia, and the U.S. Studies included a variety of pollen types and air pollutants, with little consistency between them. Health outcomes considered were all respiratory morbidity (mostly hospital admissions and emergency department visits), with the exception of one that focused on cardiopulmonary emergency department visits [101].

The studies in this category were inconsistent in their study designs and findings. For example, Anderson et al. [92] concluded that there was no evidence for synergy between air pollutants and pollen, with the exception of SO_2_ and grass pollen in children during the warm season. Chen et al. [94] also found little evidence of interactions between air pollutants and pollen but did find that several of the air pollution and pollen exposures were stronger in the cool season than in the warm season. In contrast, Goodman et al. [98] found that, in most populations, adjusting for outdoor pollen generally attenuated relative risk of hospital admissions for both ozone and PM_2.5_. Ross et al. [100] found the association between ozone and asthma medication use was increased after adjusting for aeroallergens. Cakmak et al. [93] found that there were synergistic effects on asthma hospitalization between tree pollen and increasing PM_2.5_, and between weed pollen and PM_10_.

Given that the 10 studies included inconsistent pollen types and air pollutants, with inconsistent results, we were unable to draw strong conclusions for this category. Overall, we rated the quality of the evidence as “Low” and the strength of the evidence as “Limited.” We did not upgrade the quality of the evidence since the studies reported inconsistent findings, and since studies that did find synergistic effects reported effect estimates that had low magnitudes (Table 4).

**Table 4:** Rating of the quality and strength of the evidence for studies assessing interactive effects between air pollution and pollen (*n* = 10)

**Category**	**Summary of Criteria**	**Downgrades**	**Rationale**
Initial Rating of Human Evidence = “Moderate”			
Risk of Bias	Study limitations- a substantial risk of bias across body of evidence.	-1	Downgraded because of “high” or “probably high” risk of bias for air pollution exposure assessment for six studies and “high” or “probably high” risk of bias for pollen exposure assessment for six studies.
Indirectness	Evidence was not directly comparable to the chosen population, exposure, comparator, and outcome.	0	Measured outcomes were assessed for humans in the populations for the duration of study periods, as outlined in the PECO statement.
Inconsistency	Wide variability in estimates of effect in similar populations.	0	The studies were inconsistent in pollen types and air pollutants, precluding judgment as to whether reported effect estimates would be consistent or inconsistent.
Imprecision	Studies had a small sample size and small outcome count.	0	The studies had large sample sizes with adequate samples for outcomes during study periods.
Publication Bias	Studies missing for body of evidence, resulting in an over or underestimate of true effects from exposure.	0	The studies were large studies that varied in year, data sources, and methods of statistical analysis that appeared to report outcomes found regardless of results.
**Category**	**Summary of Criteria**	**Upgrades**	**Rationale**
Large magnitude of effects	Study found confounding alone unlikely to explain association with large effect estimate as judged by reviewers.	0	Studies that reported positive associations of interactions reported effect estimates with low magnitudes.
Dose-response	Consistent relationship between dose and response in one or multiple studies, and/or exposure response across studies	0	Studies did not report a consistent relationship between dose and response.
Confounding minimizes effect	Upgraded if consideration of all plausible residual confounders or biases would underestimate the effect or suggest a spurious effect when results show no effect.	0	No evidence that residual confounders or biases would underestimate the effect or suggest a spurious effect when results show no effect
Overall Quality of Evidence		Low	The overall quality of the evidence supporting interactive effects is low.
Overall Strength of Evidence		Limited	An association was shown in a few studies between air pollution and pollen and increased outcomes, however the results were inconsistent and there was a potentially high risk of bias from the exposure assessments in several studies.

Our search only found one study that examined interactions between heat and pollen [102]. This study explored climate factors and pollen count impacts on pediatric hay fever prevalence among 91,642 children across the U.S. Hay fever prevalence was shown to increase with the second, third, and fourth quartile mean annual temperature and mean total pollen counts. This study was particularly strong given the large size and national representation of the included population. However, with only one study, we did not draw conclusions regarding the quality and strength of evidence for interactive effects between heat and pollen.

## Discussion

We conducted a systematic literature review of human population health studies to examine the evidence for synergistic effects from simultaneous exposure to air pollution, pollen, and heat, or a subset of these three risk factors. We found limited evidence for synergistic respiratory effects of air pollution, pollen, and heat; sufficient evidence for synergistic all-cause mortality, cardiovascular, and respiratory effects of air pollution and heat (particularly for ozone and particulate matter); and limited evidence for synergistic respiratory effects of air pollution and pollen. We were unable to assess evidence for pollen and heat because only one paper came up in our searches.

Overall, there was a substantially larger body of literature examining interactive effects between air pollution and heat, compared with those that included pollen as an exposure of interest. The evidence for interactive effects between air pollution and heat is further strengthened by large datasets of pollutant levels and meteorological data, including from the National, Morbidity, Mortality, and Air Pollution Study (NMMAPS) in the U.S. and the Ultrafine Particles and Health Study Group in Europe. An additional strength across all categories was that a majority of the studies had a low risk of bias for study design, with many of them using a time series design.

Though there were some strengths in the literature, we also found serious weaknesses that precluded our ability to draw strong conclusions as to the existence of interactive health effects from simultaneous exposure to these risk factors. Limitations included that all of the studies we found were short-term studies that were unable to address effects of long-term exposure. We found no cohort studies that could properly attribute exposure at an individual level and account for health outcomes that may take years to manifest. In addition, exposure measurements and metrics for air pollutants, pollen, and temperature were inconsistent and not standardized between the studies. Judging the potential bias from exposure measurement for air pollution, temperature, and pollen is difficult with only limited information available in the papers. For example, some papers did not report the number of monitoring stations used to assign exposures or the length of time for which the exposure data were collected. Recent studies of air pollution have begun using more sophisticated methods to assign exposure, such as models that use satellite remote sensing or land use variables that provide greater spatial coverage compared with ground monitors such as those run by government monitoring networks [104–106]. For pollen, the studies in this review all used pollen count as the exposure metric, which may not account for pollen potency [23]. Another limitation is that many studies were missing information about confounders that were considered, which could influence the magnitude of the associations they found. Finally, while we restricted our review to studies that looked at interaction between two of the three hazards, several studies may have treated these risk factors as mediators or effect modifiers. Future research should explore the role of these issues. Additional research should also explore effects of these risk factors on additional health outcomes, such as birth outcomes, as well as vulnerable populations, including children, the elderly, pregnant women, and people with genetic predisposition to cardiovascular and respiratory disease.

We included only heat, air pollution, and pollen in this review, as they are all conditions of the ambient air for which we judged there to be enough epidemiological literature to assess. Other important environmental drivers of disease related to the ambient air that we did not include here are occupational exposures; different types of air pollutant mixtures (including from different combustion sources and different composition of particulate matter); and exposure to airborne bacteria, viruses, molds, and fungus. In reality, people are exposed to a complex set of risk factors that remain poorly defined and explored in the literature. In addition, the chronic diseases considered affected by these risk factors are multi-factorial with heavy influence from genetic and lifestyle (e.g. diet, exercise) factors. Our literature review highlights the importance of including environmental factors in epidemiological and risk assessment studies, even if strong conclusions cannot yet be drawn from the current set of available studies.

## Conclusions

In this systematic literature review of epidemiological studies, we found evidence for synergistic effects of heat and air pollutants (particularly for ozone and particulate matter), but not for the combination of heat, air pollution, and pollen together or of air pollution and pollen or heat and pollen. Our findings support consideration of combined effects of heat and air pollution in assessing health impacts from these risk factors in the present day and in the future as climate change progresses. However, the literature is too nascent to support inclusion of interactive effects between air pollution and pollen or heat and pollen in risk assessments. Future research should continue to explore potential interactive effects of environmental exposures on human health, as people are often exposed to multiple environmental risk factors simultaneously. This is a rapidly evolving field of study, and our review and conclusions should be updated to include new evidence as it becomes available. If new evidence supports our conclusion that heat and air pollution exposure act synergistically on human health, the health impacts from climate change-driven increases in air pollution and heat exposure may be larger than previously estimated in studies that consider these risk factors individually.

## Supplementary Information

**Additional file 1.**

## Data Availability

All data are available within the article and supplemental material.

## Abbreviations

CO: Carbon monoxide
GRADE: Grading of Recommendations Assessment, Development and Evaluation
NO2: Nitrogen dioxide
O3: Ozone
PECO: Population, Exposure, Control, Outcome
PM2.5: Fine particulate matter
PM10: Coarse particulate matter
SO2: Sulfur dioxide

## References

[1] Fiore AM, Naik V, Leibensperger EM (2015). Air quality and climate connections. J Air Waste Manage Assoc.

[2] Kinney PL (2008). Climate change, air quality, and human health. Am J Prev Med.

[3] Kinney PL (2018). Interactions of climate change, air pollution, and human health. Curr Envir Health Rpt.

[4] U.S. Environmental Protection Agency. Multi-Model Framework for Quantitative Sectoral Impacts Analysis: A Technical Report for the Fourth National Climate Assessment [Internet]. U.S. Environmental Protection Agency; 2017 [cited 2017 Nov 8]. Available from: https://cfpub.epa.gov/si/si_public_record_Report.cfm?dirEntryId=335095.

[5] US Global Change Research Program. The Impacts of Climate Change on Human Health in the United States: A Scientific Assessment [Internet]. U.S. Global Change Research Program (USGCRP); 2016. Available from: 10.7930/J0R49NQX.

[6] McGeehin MA, Mirabelli M (2001). The potential impacts of climate variability and change on temperature-related morbidity and mortality in the United States. Environ Health Perspect.

[7] Melillo JM, Richmond T (T. C), Yohe GW. Climate Change Impacts in the United States: The Third National Climate Assessment [Internet]. U.S. Global Change Research Program; 2014 [cited 2020 Nov 18]. Available from: https://nca2014.globalchange.gov/downloads.

[8] Xu R, Zhao Q, Coelho MSZS, Saldiva PHN, Zoungas S, Huxley RR (2019). Association between heat exposure and hospitalization for diabetes in Brazil during 2000–2015: a Nationwide case-crossover study. Environ Health Perspect.

[9] Anderson GB, Dominici F, Wang Y, McCormack MC, Bell ML, Peng RD (2013). Heat-related emergency hospitalizations for respiratory diseases in the Medicare population. Am J Respir Crit Care Med.

[10] Mannino DM (2006). The natural history of chronic obstructive pulmonary disease. Eur Respir J.

[11] White MD (2006). Components and mechanisms of thermal hyperpnea. J Appl Physiol.

[12] Liu C, Yavar Z, Sun Q (2015). Cardiovascular response to thermoregulatory challenges. Am J Phys Heart Circ Phys.

[13] Petkova E, Bader D, Anderson G, Horton R, Knowlton K, Kinney P (2014). Heat-related mortality in a warming climate: projections for 12 U.S. cities. IJERPH..

[14] Remigio RV, Jiang C, Raimann J, Kotanko P, Usvyat L, Maddux FW (2019). Association of Extreme Heat Events with Hospital Admission or mortality among patients with end-stage renal disease. JAMA Netw Open.

[15] Reidmiller DR, Avery CW, Easterling DR, Kunkel KE, Lewis KLM, Maycock TK, et al. Impacts, Risks, and Adaptation in the United States: The Fourth National Climate Assessment, Volume II [Internet]. U.S. Global Change Research Program; 2018 [cited 2020 Nov 18]. Available from: https://nca2018.globalchange.gov/.

[16] Wu S, Mickley LJ, Leibensperger EM, Jacob DJ, Rind D, Streets DG (2008). Effects of 2000–2050 global change on ozone air quality in the United States. J Geophys Res.

[17] Integrated Science Assessment (ISA) for Particulate Matter (Final Report, Dec 2019) [Internet]. Washington, DC, U.S.: U.S. Environmental Protection Agency; 2019 p. 1967. Report No.: EPA/600/R−19/188, 2019. Available from: https://cfpub.epa.gov/ncea/isa/recordisplay.cfm?deid=347534.

[18] Integrated Science Assessment (ISA) for Ozone and Related Photochemical Oxidants (Final Report, Feb 2013) [Internet]. Washington, DC, U.S.: U.S. Environmental Protection Agency; 2013 p. 1251. Report No.: EPA/600/R-10/076F, 2013. Available from: https://cfpub.epa.gov/ncea/isa/recordisplay.cfm?deid=247492.

[19] Cosselman KE, Navas-Acien A, Kaufman JD (2015). Environmental factors in cardiovascular disease. Nat Rev Cardiol.

[20] Garcia-Menendez F, Saari RK, Monier E, Selin NE (2015). U.S. air quality and health benefits from avoided climate change under greenhouse gas mitigation. Environ Sci Technol.

[21] Ziska LH, Beggs PJ (2012). Anthropogenic climate change and allergen exposure: the role of plant biology. J Allergy Clin Immunol.

[22] D’Amato G, Rottem M, Dahl R, Blaiss M, Ridolo E, Cecchi L (2011). Climate change, migration, and allergic respiratory diseases: an update for the allergist. World Allergy Organ J.

[23] D’Amato G, Holgate ST, Pawankar R, Ledford DK, Cecchi L, Al-Ahmad M (2015). Meteorological conditions, climate change, new emerging factors, and asthma and related allergic disorders. A statement of the World Allergy Organization. World Allergy Organization J.

[24] Gilles S, Blume C, Wimmer M, Damialis A, Meulenbroek L, Gökkaya M (2020). Pollen exposure weakens innate defense against respiratory viruses. Allergy..

[25] Weinberger SE, Cockrill BA, Mandel J (2019). Principles of pulmonary medicine.

[26] Weichenthal S, Lavigne E, Villeneuve PJ, Reeves F (2016). Airborne pollen concentrations and emergency room visits for myocardial infarction: a multicity case-crossover study in Ontario, Canada. Am J Epidemiol.

[27] Peel JL, Tolbert PE, Klein M, Metzger KB, Flanders WD, Todd K (2005). Ambient Air Pollution and Respiratory Emergency Department Visits. Epidemiology.

[28] Rajagopalan S, Al-Kindi SG, Brook RD (2018). Air pollution and cardiovascular disease. J Am Coll Cardiol.

[29] Soneja S, Jiang C, Fisher J, Upperman CR, Mitchell C, Sapkota A (2016). Exposure to extreme heat and precipitation events associated with increased risk of hospitalization for asthma in Maryland, USA. Environ Health.

[30] Baldacci S, Maio S, Cerrai S, Sarno G, Baïz N, Simoni M (2015). Allergy and asthma: effects of the exposure to particulate matter and biological allergens. Respir Med.

[31] Sedghy F, Varasteh A-R, Sankian M, Moghadam M (2018). Interaction between air pollutants and pollen grains: the role on the rising trend in allergy. Rep Biochem Mol Biol.

[32] De Sario M, Katsouyanni K, Michelozzi P (2013). Climate change, extreme weather events, air pollution and respiratory health in Europe. Eur Respir J.

[33] Cayrol C, Duval A, Schmitt P, Roga S, Camus M, Stella A (2018). Environmental allergens induce allergic inflammation through proteolytic maturation of IL-33. Nat Immunol.

[34] Anenberg SC, Weinberger KR, Henry R, Neumann JE, Crimmins A, Fann N (2017). Impacts of oak pollen on allergic asthma in the United States and potential influence of future climate change. GeoHealth..

[35] Achakulwisut P, Mickley LJ, Anenberg SC (2018). Drought-sensitivity of fine dust in the US southwest: implications for air quality and public health under future climate change. Environ Res Lett.

[36] Achakulwisut P, Anenberg SC, Neumann JE, Penn SL, Weiss N, Crimmins A (2019). Effects of increasing aridity on ambient dust and public health in the U.S. southwest under climate change. GeoHealth..

[37] Fann N, Nolte CG, Dolwick P, Spero TL, Brown AC, Phillips S (2015). The geographic distribution and economic value of climate change-related ozone health impacts in the United States in 2030. J Air Waste Manage Assoc.

[38] Ford B, Val Martin M, Zelasky SE, Fischer EV, Anenberg SC, Heald CL (2018). Future fire Impacts on smoke concentrations, visibility, and health in the contiguous United States. GeoHealth..

[39] Li T, Horton RM, Kinney PL (2013). Projections of seasonal patterns in temperature- related deaths for Manhattan, New York. Nature Clim Change.

[40] Neumann JE, Anenberg SC, Weinberger KR, Amend M, Gulati S, Crimmins A (2019). Estimates of present and future asthma emergency department visits associated with exposure to oak, birch, and grass pollen in the United States. GeoHealth..

[41] Martinich J, Crimmins A (2019). Climate damages and adaptation potential across diverse sectors of the United States. Nat Clim Chang.

[42] Woodruff TJ, Sutton P (2011). The navigation guide work group. An evidence-based medicine methodology to bridge the gap between clinical and environmental health sciences. Health Aff.

[43] Higgins JPT, Green S. Cochrane handbook for systematic reviews of interventions. [Internet]. 5.1.0. Hoboken, N.J.: Wiley; 2011 [cited 2020 Nov 17]. 672 p. Available from: http://public.ebookcentral.proquest.com/choice/publicfullrecord.aspx?p=4041340.

[44] Dijkers M. Introducing GRADE: a systematic approach to rating evidence in systematic reviews and to guideline development [Internet]. Austin, TX, USA: Center on Knowledge Translation for Disability and Rehabilitation Research; 2013 p. 9. Report No.: 5.1.0. Available from: https://ktdrr.org/products/update/v1n5/dijkers_grade_ktupdatev1n5.pdf.

[45] Schünemann H, Brożek J, Guyatt G, Oxman A. GRADE Handbook [Internet]. 2013. Available from: https://gdt.gradepro.org/app/handbook/handbook.html.

[46] Johnson PI, Sutton P, Atchley DS, Koustas E, Lam J, Sen S (2014). The navigation guide—evidence-based medicine meets environmental health: systematic review of human evidence for PFOA effects on fetal growth. Environ Health Perspect.

[47] Hebbern C, Cakmak S (2015). Synoptic weather types and aeroallergens modify the effect of air pollution on hospitalisations for asthma hospitalisations in Canadian cities. Environ Pollut.

[48] Makra L, Puskás J, Matyasovszky I, Csépe Z, Lelovics E, Bálint B (2015). Weather elements, chemical air pollutants and airborne pollen influencing asthma emergency room visits in Szeged, Hungary: performance of two objective weather classifications. Int J Biometeorol.

[49] Matyasovszky I, Makra L, Bálint B, Guba Z, Sümeghy Z (2011). Multivariate analysis of respiratory problems and their connection with meteorological parameters and the main biological and chemical air pollutants. Atmos Environ.

[50] Mazenq J, Dubus J-C, Gaudart J, Charpin D, Nougairede A, Viudes G (2017). Air pollution and children’s asthma-related emergency hospital visits in southeastern France. Eur J Pediatr.

[51] Mireku N, Wang Y, Ager J, Reddy RC, Baptist AP (2009). Changes in weather and the effects on pediatric asthma exacerbations. Ann Allergy Asthma Immunol.

[52] Witonsky J, Abraham R, Toh J, Desai T, Shum M, Rosenstreich D (2019). The association of environmental, meteorological, and pollen count variables with asthma-related emergency department visits and hospitalizations in the Bronx. J Asthma.

[53] Analitis A, Michelozzi P, D’Ippoliti D (2014). de’Donato F, Menne B, Matthies F, et al. effects of heat waves on mortality: effect modification and confounding by air pollutants. Epidemiology..

[54] Analitis A, de’ Donato F, Scortichini M, Lanki T, Basagana X, Ballester F (2018). Synergistic Effects of Ambient Temperature and Air Pollution on Health in Europe: Results from the PHASE Project. IJERPH.

[55] Breitner S, Wolf K, Devlin RB, Diaz-Sanchez D, Peters A, Schneider A (2014). Short-term effects of air temperature on mortality and effect modification by air pollution in three cities of Bavaria, Germany: a time-series analysis. Sci Total Environ.

[56] Cheng Y, Kan H (2012). Effect of the interaction between outdoor air pollution and extreme temperature on daily mortality in Shanghai. China Journal of Epidemiology.

[57] Li G, Zhou M, Cai Y, Zhang Y, Pan X (2011). Does temperature enhance acute mortality effects of ambient particle pollution in Tianjin City, China. Science of The Total Environment.

[58] Li L, Yang J, Guo C, Chen P-Y, Ou C-Q, Guo Y (2015). Particulate matter modifies the magnitude and time course of the non-linear temperature-mortality association. Environ Pollut.

[59] Lokys HL, Junk J, Krein A (2018). Short-term effects of air quality and thermal stress on non-accidental morbidity—a multivariate meta-analysis comparing indices to single measures. Int J Biometeorol.

[60] Burkart K, Canário P, Breitner S, Schneider A, Scherber K, Andrade H (2013). Interactive short-term effects of equivalent temperature and air pollution on human mortality in Berlin and Lisbon. Environ Pollut.

[61] Chen K, Wolf K, Breitner S, Gasparrini A, Stafoggia M, Samoli E (2018). Two-way effect modifications of air pollution and air temperature on total natural and cardiovascular mortality in eight European urban areas. Environ Int.

[62] Chen K, Wolf K, Hampel R, Stafoggia M, Breitner S, Cyrys J, et al. Does temperature-confounding control influence the modifying effect of air temperature in ozone–mortality associations?: Environmental Epidemiology. 2018 Mar;2(1):e008.

[63] Dear K, Ranmuthugala G, Kjellström T, Skinner C, Hanigan I (2005). Effects of temperature and ozone on daily mortality during the august 2003 heat wave in France. Archives of Environmental & Occupational Health..

[64] Filleul L, Cassadou S, Médina S, Fabres P, Lefranc A, Eilstein D (2006). The relation between temperature, ozone, and mortality in nine French cities during the heat wave of 2003. Environ Health Perspect.

[65] Jhun I, Fann N, Zanobetti A, Hubbell B (2014). Effect modification of ozone-related mortality risks by temperature in 97 US cities. Environ Int.

[66] Kim SE, Lim Y-H, Kim H (2015). Temperature modifies the association between particulate air pollution and mortality: a multi-city study in South Korea. Sci Total Environ.

[67] Liu T, Zeng W, Lin H, Rutherford S, Xiao J, Li X (2016). Tempo-spatial variations of ambient ozone-mortality associations in the USA: results from the NMMAPS data. IJERPH..

[68] Meng X, Zhang Y, Zhao Z, Duan X, Xu X, Kan H (2012). Temperature modifies the acute effect of particulate air pollution on mortality in eight Chinese cities. Sci Total Environ.

[69] Moolgavkar SH (2003). Air pollution and daily mortality in two U.S. counties: season-specific analyses and exposure-response relationships. Inhal Toxicol.

[70] Park AK, Hong YC, Kim H (2011). Effect of changes in season and temperature on mortality associated with air pollution in Seoul, Korea. J Epidemiol Community Health.

[71] Pattenden S, Armstrong B, Milojevic A, Heal MR, Chalabi Z, Doherty R (2010). Ozone, heat and mortality: acute effects in 15 British conurbations. Occup Environ Med.

[72] Peng RD, Samoli E, Pham L, Dominici F, Touloumi G, Ramsay T (2013). Acute effects of ambient ozone on mortality in Europe and North America: results from the APHENA study. Air Qual Atmos Health.

[73] Rainham DGC, Smoyer-Tomic KE, Sheridan SC, Burnett RT (2005). Synoptic weather patterns and modification of the association between air pollution and human mortality. Int J Environ Health Res.

[74] Scortichini M, De Sario M, de Donato F, Davoli M, Michelozzi P, Stafoggia M (2018). Short-Term Effects of Heat on Mortality and Effect Modification by Air Pollution in 25 Italian Cities. IJERPH.

[75] Shaposhnikov D, Revich B, Bellander T, Bedada GB, Bottai M, Kharkova T (2014). Mortality Related to Air Pollution with the Moscow Heat Wave and Wildfire of 2010. Epidemiology.

[76] Stafoggia M, Schwartz J, Forastiere F, Perucci CA (2008). Does temperature modify the association between air pollution and mortality? A multicity case-crossover analysis in Italy. Am J Epidemiol.

[77] Sun S, Cao P, Chan K-P, Tsang H, Wong C-M, Thach T-Q (2015). Temperature as a modifier of the effects of fine particulate matter on acute mortality in Hong Kong. Environ Pollut.

[78] Vanos JK, Cakmak S, Kalkstein LS, Yagouti A (2015). Association of weather and air pollution interactions on daily mortality in 12 Canadian cities. Air Qual Atmos Health.

[79] Wilson A, Rappold AG, Neas LM, Reich BJ (2014). Modeling the effect of temperature on ozone-related mortality. Ann Appl Stat.

[80] Zhang Y, Huang W, London SJ, Song G, Chen G, Jiang L (2006). Ozone and daily mortality in Shanghai, China. Environmental Health Perspectives.

[81] Ding P-H, Wang G-S, Guo Y-L, Chang S-C, Wan G-H (2017). Urban air pollution and meteorological factors affect emergency department visits of elderly patients with chronic obstructive pulmonary disease in Taiwan. Environ Pollut.

[82] Jo E-J, Lee W-S, Jo H-Y, Kim C-H, Eom J-S, Mok J-H (2017). Effects of particulate matter on respiratory disease and the impact of meteorological factors in Busan. Korea Respiratory Medicine.

[83] Kunikullaya KU, Vijayaraghava A, Asha P, Kunnavil R, MuraliMohan BV. Meteorological parameters and pollutants on asthma exacerbation in Bangalore, India – an ecological retrospective time-series study. Journal of Basic and Clinical Physiology and Pharmacology [Internet]. 2017 Jan 1 [cited 2020 Nov 17];28(2). Available from: https://www.degruyter.com/doi/10.1515/jbcpp-2016-0074. 10.1515/jbcpp-2016-0074 28076315

[84] Lam HC, Li AM, Chan EY, Goggins WB (2016). The short-term association between asthma hospitalisations, ambient temperature, other meteorological factors and air pollutants in Hong Kong: a time-series study. Thorax..

[85] Mirabelli MC, Vaidyanathan A, Flanders WD, Qin X, Outdoor PM GP (2016). Ambient Air Temperature, and Asthma Symptoms in the Past 14 Days among Adults with Active Asthma. Environ Health Perspect.

[86] Qiu H, Tan K, Long F, Wang L, Yu H, Deng R (2018). The burden of COPD morbidity attributable to the interaction between ambient air pollution and temperature in Chengdu, China. IJERPH.

[87] Winquist A, Kirrane E, Klein M, Strickland M, Darrow LA, Sarnat SE (2014). Joint Effects of Ambient Air Pollutants on Pediatric Asthma Emergency Department Visits in Atlanta, 1998–2004. Epidemiology.

[88] Lee H, Myung W, Cheong H-K, Yi S-M, Hong Y-C, Cho S-I (2018). Ambient air pollution exposure and risk of migraine: synergistic effect with high temperature. Environ Int.

[89] Luo K, Li R, Wang Z, Zhang R, Xu Q (2017). Effect modification of the association between temperature variability and daily cardiovascular mortality by air pollutants in three Chinese cities. Environ Pollut.

[90] Ren C, Williams GM, Morawska L, Mengersen K, Tong S (2008). Ozone modifies associations between temperature and cardiovascular mortality: analysis of the NMMAPS data. Occup Environ Med.

[91] Ren C, Williams GM, Mengersen K, Morawska L, Tong S (2009). Temperature enhanced effects of ozone on cardiovascular mortality in 95 large US communities, 1987–2000: assessment using the NMMAPS data. Archives of Environmental & Occupational Health.

[92] Anderson HR, de Leon AP, Bland JM, Bower JS, Emberlin J, Strachan DP (1998). Air pollution, pollens, and daily admissions for asthma in London 1987-92. Thorax..

[93] Cakmak S, Dales RE, Coates F (2012). Does air pollution increase the effect of aeroallergens on hospitalization for asthma?. J Allergy Clin Immunol.

[94] Chen K, Glonek G, Hansen A, Williams S, Tuke J, Salter A (2016). The effects of air pollution on asthma hospital admissions in Adelaide, South Australia, 2003-2013: time-series and case-crossover analyses. Clin Exp Allergy.

[95] Cirera L, García-Marcos L, Giménez J, Moreno-Grau S, Tobías A, Pérez-Fernández V (2012). Daily effects of air pollutants and pollen types on asthma and COPD hospital emergency visits in the industrial and Mediterranean Spanish city of Cartagena. Allergol Immunopathol.

[96] Galán I, Tobías A, Banegas JR, Aránguez E (2003). Short-term effects of air pollution on daily asthma emergency room admissions. Eur Respir J.

[97] Gleason JA, Bielory L, Fagliano JA (2014). Associations between ozone, PM2.5, and four pollen types on emergency department pediatric asthma events during the warm season in New Jersey: a case-crossover study. Environ Res.

[98] Goodman JE, Loftus CT, Liu X, Zu K (2017). Impact of respiratory infections, outdoor pollen, and socioeconomic status on associations between air pollutants and pediatric asthma hospital admissions. Larcombe A, editor. PLoS ONE.

[99] Krmpotic D, Luzar-Stiffler V, Rakusic N, Stipic Markovic A, Hrga I, Pavlovic M (2011). Effects of traffic air pollution and hornbeam pollen on adult asthma hospitalizations in Zagreb. Int Arch Allergy Immunol.

[100] Ross MA, Persky VW, Scheff PA, Chung J, Curtis L, Ramakrishnan V (2002). Effect of ozone and aeroallergens on the respiratory health of asthmatics. Archives of Environmental Health: An International Journal.

[101] Stieb DM, Beveridge RC, Brook JR, Smith-Doiron M, Burnett RT, Dales RE (2000). Air pollution, aeroallergens and cardiorespiratory emergency department visits in Saint John, Canada. J Expo Sci Environ Epidemiol.

[102] Silverberg JI, Braunstein M, Lee-Wong M (2015). Association between climate factors, pollen counts, and childhood hay fever prevalence in the United States. Journal of Allergy and Clinical Immunology.

[103] Adelborg K, Szépligeti SK, Holland-Bill L, Ehrenstein V, Horváth-Puhó E, Henderson VW (2018). Migraine and risk of cardiovascular diseases: Danish population based matched cohort study. BMJ..

[104] Jerrett M, Turner MC, Beckerman BS, Pope CA, van Donkelaar A, Martin RV (2017). Comparing the health effects of ambient particulate matter estimated using ground-based versus remote sensing exposure estimates. Environ Health Perspect.

[105] Vodonos A, Awad YA, Schwartz J (2018). The concentration-response between long-term PM2.5 exposure and mortality; a meta-regression approach. Environ Res.

[106] Di Q, Wang Y, Zanobetti A, Wang Y, Koutrakis P, Choirat C (2017). Air pollution and mortality in the Medicare population. N Engl J Med.

