# Supplementary material for: Synergistic health effects of air pollution, temperature, and pollen exposure: a systematic review of epidemiological evidence
Source: Environ Health. 2020 Dec 7;19:130. doi: 10.1186/s12940-020-00681-z (PMC7720572; doi:10.1186/s12940-020-00681-z)
Supplement: Supplementary file 1 — Additional file 1. [file 12940_2020_681_MOESM1_ESM.docx]

**Synergistic health effects of air pollution, temperature, and pollen exposure: A systematic review of epidemiological evidence**

Susan C. Anenberg, Shannon Haines, Elizabeth Wang, Nicholas Nassikas, Patrick L. Kinney

**Supplementary Tables**

Table S1. Search terms used and the number of records returned from each database.

| Database (n=results) | Search Terms |
| --- | --- |
| Scopus (n=418) | Exposure: (“heat” or “temperature”) and (“air pollution” or “air quality” or “dust” or “no2” or “so2” or “particulate matter” or “ozone” or “multi pollutant”) and (“pollen” or “aeroallergens”) Outcome: (“cardiovascular” or “respiratory” or “mortality” or “asthma” or “allergies”) |
| PubMed (n=513) | Exposure: (“heat” or “temperature”) and (“air pollution” or “air quality” or “dust” or “NO2” or “SO2” or “particulate matter” or “ozone” or “multi pollutant”) and (“pollen” or “aeroallergens”) Outcome: (“cardiovascular” or “respiratory” or “mortality” or “asthma” or “allergies”) |
| Google Scholar (n=45) | Exposure: (“heat” or “temperature”) and (“air pollution” or “air quality” or “dust” or “NO2” or “SO2” or “particulate matter” or “ozone” or “multi pollutant”) and (“pollen” or “aeroallergens”) Outcome: (“cardiovascular” or “respiratory” or “mortality” or “asthma” or “allergies”) |
| Proquest (n=754) | Exposure: (“dust” or “air pollution” or “air quality” or “particulate matter” or “PM2.5” or “ozone” or “NO2”) and (“heat” or “temperature”) and (“pollen” or “aeroallergens”) and ("joint effects" or "multipollutant model") Outcome: (“cardiovascular disease” or “respiratory disease” or “mortality” or “asthma” or “stroke”) |

Tables S2 through S57. Risk of bias determination and rationale for each study.

| Table S2. Analitis et al 2014 | |
| --- | --- |
| Study Design | Rated as low risk of bias due to time series design. |
| Exposure Assessment (Air Pollution) | Rated as probably low risk of bias because concentrations of SO_2_, PM_10_, NO_2_, O_3_, and CO were obtained from an unknown number of monitoring sites in each city. |
| Exposure Assessment (Temperature) | Rated as probably low risk of bias because daily air temperature data were obtained from the EuroHEAT project. |
| Exposure Assessment (Pollen) | Pollen is not assessed in this study. |
| Detection of Outcome | Rated as low risk of bias because the outcome is classified using ICD-9 codes for deaths from natural causes, cardiovascular diseases, respiratory causes, and cerebrovascular causes. |
| Reporting | Rated as low risk of bias because all outcomes are reported. |
| Conflict of Interest | Rated as probably low risk of bias because the authors do not mention conflict of interest. |
|  |  |
| Table S3. Analitis et al 2018 | |
| Study Design | Rated as low risk of bias due to time series design. |
| Exposure Assessment (Air Pollution) | Rated as probably low risk because concentrations of PM_10_, O_3_, NO_2_ were obtained from the urban monitoring network of each city but not at an individual level. |
| Exposure Assessment (Temperature) | Rated as probably low risk of bias because year-round daily air temperature data were obtained from the PHASE project. |
| Exposure Assessment (Pollen) | Pollen is not assessed in this study. |
| Detection of Outcome | Rated as low risk of bias because the outcome is classified using ICD codes from the official registries of each city. |
| Reporting | Rated as low risk of bias because all outcomes are reported. |
| Conflict of Interest | Rated as low risk of bias because the authors declare no conflict of interest. |
|  |  |
| Table S4. Anderson et al 1998 | |
| Study Design | Rated as low risk of bias due to time series design. |
| Exposure Assessment (Air Pollution) | Rated as probably high risk of bias because concentrations of O_3_, NO_2_, SO_2_, and black smoke were measured at between one to four monitoring sites at in the city. |
| Exposure Assessment (Temperature) | Rated as probably low risk of bias because mean 24 hour temperatures are calculated from data obtained from central London but no further details are given. |
| Exposure Assessment (Pollen) | Rated as high risk of bias because daily counts of pollens were averaged using an unspecified spore trap but only from one monitoring site in the city. |
| Detection of Outcome | Rated as low risk of bias because the outcome was measured by ICD codes for ER admissions for asthma. |
| Reporting | Rated as low risk of bias because all outcomes are reported. |
| Conflict of Interest | Rated as probably low risk of bias because the authors do not mention conflict of interest. |
|  |  |
| Table S5. Breitner et al 2014 | |
| Study Design | Rated as low risk of bias due to time series design. |
| Exposure Assessment (Air Pollution) | Rated as probably high risk of bias because concentrations of PM_10_ and ozone are measured at between one to five monitoring sites in each city. |
| Exposure Assessment (Temperature) | Rated as low risk of bias because mean 24 hour temperatures were calculated from hourly data from the German Weather Service and Bavarian Environment Agency. |
| Exposure Assessment (Pollen) | Pollen is not assessed in this study. |
| Detection of Outcome | Rated as low risk of bias because the outcome is classified using ICD-9 and ICD-10 codes for mortality. |
| Reporting | Rated as low risk of bias because all outcomes are reported. |
| Conflict of Interest | Rated as low risk of bias because the authors declare no conflict of interest. |
|  |  |
| Table S6. Burkart et al 2013 | |
| Study Design | Rated as low risk of bias due to time series design. |
| Exposure Assessment (Air Pollution) | Rated as probably high risk of bias because hourly concentrations of PM_10_ and O_3_ were measured at three monitoring sites in each city but some measurements are inconsistent and incomplete. |
| Exposure Assessment (Temperature) | Rated as low risk of bias because hourly temperature data for both cities were obtained from the German Weather Service and National Climatic Data Centre. |
| Exposure Assessment (Pollen) | Pollen is not assessed in this study. |
| Detection of Outcome | Rated as probably low risk of bias because daily death counts are obtained from state/national statistical institutes but it is unclear if ICD codes were included to validate mortalities. |
| Reporting | Rated as low risk of bias because all outcomes are reported. |
| Conflict of Interest | Rated as probably low risk of bias because the authors do not mention conflict of interest. |
|  |  |
| Table S7. Cakmak et al 2012 | |
| Study Design | Rated as low risk of bias due to time series design. |
| Exposure Assessment (Air Pollution) | Rated as probably low risk of bias because daily concentrations of SO_2_, NO_2_, CO, PM_10_, and PM_2.5_ were obtained National Air Pollution Monitoring System. but not at an individual level. |
| Exposure Assessment (Temperature) | Rated as low risk of bias because mean 24 hour temperature data were obtained from Environment Canada and the National Air Pollution Monitoring System. |
| Exposure Assessment (Pollen) | Rated as probably low risk of bias because concentrations of aeroallergens are measured using a standardized and reliable method across cities but not at an individual level. |
| Detection of Outcome | Rated as low risk of bias because the outcome was measured using ICD codes for hospital admissions for asthma. |
| Reporting | Rated as low risk of bias because all outcomes are reported. |
| Conflict of Interest | Rated as low risk of bias because the authors declare no conflict of interest. |
|  |  |
| Table S8. Chen et al 2016 | |
| Study Design | Rated as low risk of bias due to time series and case crossover design. |
| Exposure Assessment (Air Pollution) | Rated as high risk of bias because concentrations of PM_10_, PM_2.5_, NO_2_ and ozone were measured from a single monitoring site in a suburb of the city. |
| Exposure Assessment (Temperature) | Rated as low risk of bias because daily average ambient air temperature data were obtained from the Australian Bureau of Meteorology. |
| Exposure Assessment (Pollen) | Rated as probably low risk of bias because data for multiple pollens were obtained from two organizations using Hirst volumetric spore traps but not at an individual level. |
| Detection of Outcome | Rated as low risk of bias because the outcome is measured using ICD-10 codes for asthma hospital admissions. |
| Reporting | Rated as low risk of bias because all outcomes are reported. |
| Conflict of Interest | Rated as low risk of bias because the authors declare no conflict of interest. |
|  |  |
| Table S9. Chen et al 2018 (Environmental Epidemiology) | |
| Study Design | Rated as low risk of bias due to time series design. |
| Exposure Assessment (Air Pollution) | Rated as probably low risk of bias because concentrations of ozone, PM_10_, and NO_2_ were obtained for all cities using multiple local monitors but not at an individual level. |
| Exposure Assessment (Temperature) | Rated as probably low risk of bias because daily mean temperature data were obtained from local or airport meteorological stations. |
| Exposure Assessment (Pollen) | Pollen is not assessed in this study. |
| Detection of Outcome | Rated as low risk of bias because the outcome is classified using ICD-9 and ICD-10 codes for mortality. |
| Reporting | Rated as low risk of bias because all outcomes are reported. |
| Conflict of Interest | Rated as low risk of bias because the authors declare no conflict of interest. |
|  |  |
| Table S10. Chen et al 2018 (Environmental International) | |
| Study Design | Rated as low risk of bias due to time series design. |
| Exposure Assessment (Air Pollution) | Rated as probably low risk of bias because PNC, PM_2.5_, PM_10_, and O_3_ were measured from multiple local monitoring stations in each city but not at an individual level. |
| Exposure Assessment (Temperature) | Rated as probably low risk of bias because daily mean air temperature data were obtained from local meteorological services or airport meteorological networks. |
| Exposure Assessment (Pollen) | Pollen is not assessed in this study. |
| Detection of Outcome | Rated as low risk of bias because the outcome is classified using ICD-9 and ICD-10 codes for mortality. |
| Reporting | Rated as low risk of bias because all outcomes are reported. |
| Conflict of Interest | Rated as low risk of bias because the authors declare no conflict of interest. |
|  |  |
| Table S11. Cirera et al 2012 | |
| Study Design | Rated as low risk of bias because of time series design. |
| Exposure Assessment (Air Pollution) | Rated as probably high risk of bias because daily levels of TSP, SO_2_, NO_2_, and O_3_ were measured by three monitors or less in varying locations. |
| Exposure Assessment (Temperature) | Rated as low risk of bias because daily hour values of minimum and maximum temperature were obtained from the Spanish National Agency of Meteorology. |
| Exposure Assessment (Pollen) | Rated as probably high risk of bias because the Hirst method sampler was used to measure pollen concentrations from a central location but not at an individual level. |
| Detection of Outcome | Rated as probably low risk of bias because the outcome is classified by asthma and COPD cases from ER registries but it is unclear if ICD codes were used. |
| Reporting | Rated as low risk of bias because all outcomes are reported. |
| Conflict of Interest | Rated as low risk of bias because the authors declare no conflict of interest. |
|  |  |
| Table S12. Dear et al 2005 | |
| Study Design | Rated as low risk of bias due to time series design. |
| Exposure Assessment (Air Pollution) | Rated as probably high risk of bias because only daily peak levels of photochemical ozone are estimated using PREV’AIR models. |
| Exposure Assessment (Temperature) | Rated as probably low risk of bias because daily minimum and maximum temperatures were obtained from a study conducted by the French Institute for Health Surveillance. |
| Exposure Assessment (Pollen) | Pollen is not assessed in this study. |
| Detection of Outcome | Rated as probably low risk of bias because the outcome is classified by number of deaths in each city but it is unclear if ICD codes were used. |
| Reporting | Rated as low risk of bias because all outcomes are reported. |
| Conflict of Interest | Rated as probably low risk of bias because the authors do not discuss conflict of interest. |
|  |  |
| Table S13. Ding et al 2017 | |
| Study Design | Rated as low risk of bias due to time series design. |
| Exposure Assessment (Air Pollution) | Rated as probably low risk of bias because hourly concentrations of PM_10_, O_3_, SO_2_, CO and NO_2_ were measured at 11 monitoring stations in the area. |
| Exposure Assessment (Temperature) | Rated low risk of bias because daily temperature data were obtained from the two Central Weather Bureau stations in the area. |
| Exposure Assessment (Pollen) | Pollen is not assessed in this study. |
| Detection of Outcome | Rated as low risk of bias because the outcome is classified by ICD-9 codes for COPD-associated ED visits. |
| Reporting | Rated as low risk of bias because all outcomes are reported. |
| Conflict of Interest | Rated as low risk of bias because the authors declare no conflict of interest. |
|  |  |
| Table S14. Filleul et al 2006 | |
| Study Design | Rated as low risk of bias due to time series design. |
| Exposure Assessment (Air Pollution) | Rated as probably low risk of bias because concentrations of O_3_ were measured by local air monitoring networks, which are nationally standardized. |
| Exposure Assessment (Temperature) | Rated as low risk of bias because daily minimum and maximum temperatures were obtained from Météo-France. |
| Exposure Assessment (Pollen) | Pollen is not assessed in this study. |
| Detection of Outcome | Rated as probably low risk of bias because the outcome is classified using mortality data from the National Institutes of Statistics and Economic Studies but it is unclear if ICD codes were used. |
| Reporting | Rated as low risk of bias because all outcomes are reported. |
| Conflict of Interest | Rated low risk of bias because the authors declare no competing financial interests. |
|  |  |
| Table S15. Galan et al 2003 | |
| Study Design | Rated as low risk of bias due to time series design. |
| Exposure Assessment (Air Pollution) | Rated as probably high risk of bias because concentrations of PM_10_, SO_2_, and NO_2_ were measured at 13 to 15 monitoring stations but O_3_ was only measured at two. |
| Exposure Assessment (Temperature) | Rated as low risk of bias because daily mean temperature data were obtained from the Barajas Meteorological Observatory. |
| Exposure Assessment (Pollen) | Rated as probably low risk of bias because pollen data were measured using a network of 10 monitoring stations in the region. |
| Detection of Outcome | Rated as low risk of bias because the outcome is classified using ICD codes for asthma ER admissions. |
| Reporting | Rated as low risk of bias because all outcomes are reported. |
| Conflict of Interest | Rated as probably low risk of bias because the authors do not discuss conflict of interest. |
|  |  |
| Table S16. Gleason et al 2014 | |
| Study Design | Rated as low risk of bias due to case crossover design. |
| Exposure Assessment (Air Pollution) | Rated as probably low risk of bias because concentrations of PM_2.5_ and ozone were measured at 20 and 15 monitoring stations, respectively. |
| Exposure Assessment (Temperature) | Rated as low risk of bias because daily mean temperatures were obtained from the New Jersey State Climatologist using 26 weather stations. |
| Exposure Assessment (Pollen) | Rated as probably high risk of bias because pollen data was measured at only one of two monitoring stations in the state. |
| Detection of Outcome | Rated as low risk of bias because the outcome is classified using ICD-9 codes for ED visits for children with a primary diagnosis of asthma. |
| Reporting | Rated as low risk of bias because all outcomes are reported. |
| Conflict of Interest | Rated as low risk of bias because the authors declare no competing interests. |
|  |  |
| Table S17. Goodman et al 2017 | |
| Study Design | Rated as low risk of bias due to time series design. |
| Exposure Assessment (Air Pollution) | Rated as probably low risk of bias because concentrations of ozone and PM_2.5_ were obtained from multiple monitoring stations of the US EPA Air Quality System. |
| Exposure Assessment (Temperature) | Rated as probably low risk of bias because hourly weather data were obtained from the National Centers for Environmental Information. |
| Exposure Assessment (Pollen) | Rated as high risk of bias because outdoor pollen concentrations were obtained from one monitoring center and data were missing for several days and months. |
| Detection of Outcome | Rated as low risk of bias because the outcome is classified using ICD-9 codes for asthma hospital admissions. |
| Reporting | Rated as low risk of bias because all outcomes are reported. |
| Conflict of Interest | Rated as probably low risk of bias because funding for the study is provided by ExxonMobil Biomedical Sciences (a commercial company). |
|  |  |
| Table S18. Hebbern et al 2015 | |
| Study Design | Rated as low risk of bias due to time series design. |
| Exposure Assessment (Air Pollution) | Rated as probably low risk of bias because concentrations of O_3_, CO, NO_2_, SO_2_, PM_10_, and PM_2.5_ were obtained from the National Air Pollution Surveillance Network for each city. |
| Exposure Assessment (Temperature) | Rated as low risk of bias because the temperature data for each city used for the Spatial Synoptic Classification system was obtained from the Meteorological Service of Canada. |
| Exposure Assessment (Pollen) | Rated as probably low risk of bias because pollen data were collected by Aerobiology Research Laboratories using a standardized method across cities. |
| Detection of Outcome | Rated as low risk of bias because the outcome was classified using ICD-10 codes for asthma hospital admissions. |
| Reporting | Rated as low risk of bias because all outcomes are reported. |
| Conflict of Interest | Rated as low risk of bias because the authors declare no competing financial interests. |
|  |  |
| Table S19. Jhun et al 2014 | |
| Study Design | Rated as low risk of bias due to time series design. |
| Exposure Assessment (Air Pollution) | Rated as probably high risk of bias because only ozone concentrations are obtained through the NMMAPS database and other pollutants are not controlled for. |
| Exposure Assessment (Temperature) | Rated as low risk of bias because 24 hour temperature data were collected from the NMMAPS database. |
| Exposure Assessment (Pollen) | Pollen was not assessed in this study. |
| Detection of Outcome | Rated as probably low risk of bias because the outcome is measured by cause-specific deaths but it is unclear if ICD codes were used. |
| Reporting | Rated as low risk of bias because all outcomes are reported. |
| Conflict of Interest | Rated as probably low risk of bias because the authors do not discuss conflict of interest. |
|  |  |
| Table S20. Jo et al 2017 | |
| Study Design | Rated as low risk of bias due to time series design. |
| Exposure Assessment (Air Pollution) | Rated as probably low risk of bias because hourly concentrations of PM_10_ and PM_2.5_ were obtained from 19 monitoring stations in Busan. |
| Exposure Assessment (Temperature) | Rated as probably low risk of bias because temperature data were obtained from the Korean Meteorological Administration. |
| Exposure Assessment (Pollen) | Pollen is not assessed in this study. |
| Detection of Outcome | Rated as low risk of bias because the outcome is measured by hospital admissions for respiratory diseases (acute bronchitis, allergic rhinitis, and asthma) using ICD-10 codes. |
| Reporting | Rated as low risk of bias because all outcomes are reported. |
| Conflict of Interest | Rated as low risk of bias because the authors declare no conflict of interest. |
|  |  |
| Table S21. Kim et al 2015 | |
| Study Design | Rated as low risk of bias due to time series design. |
| Exposure Assessment (Air Pollution) | Rated as probably high risk of bias because concentrations of PM_10_ were measured at 88 monitoring stations but other pollutants were not controlled for. |
| Exposure Assessment (Temperature) | Rated as low risk of bias because daily mean temperature data were collected from the Korean Meteorological Office. |
| Exposure Assessment (Pollen) | Pollen was not assessed in this study. |
| Detection of Outcome | Rated as low risk of bias because the outcome was classified using ICD-10 codes for non-accidental mortality. |
| Reporting | Rated as low risk of bias because all outcomes are reported. |
| Conflict of Interest | Rated as low risk of bias because the authors declare no competing financial interests. |
|  |  |
| Table S22. Krmpotic et al 2011 | |
| Study Design | Rated as low risk of bias due to time series design. |
| Exposure Assessment (Air Pollution) | Rated as probably high risk of bias because daily averages of NO_2_, PM_10_, and CO were measured at four monitoring stations in the city. |
| Exposure Assessment (Temperature) | Rated as low risk of bias because daily mean temperature data were obtained from two monitoring stations of the Meteorological and Hydrological Service of Croatia. |
| Exposure Assessment (Pollen) | Rated as probably high risk of bias because daily pollen concentrations were obtained but it is unclear how many monitoring stations were used. |
| Detection of Outcome | Rated as low risk of bias because the outcome was classified using ICD-10 codes of asthma hospitalizations. |
| Reporting | Rated as low risk of bias because all outcomes are reported. |
| Conflict of Interest | Rated as probably low risk of bias because the authors do not discuss conflict of interest. |
|  |  |
| Table S23. Kunikullaya et al 2017 | |
| Study Design | Rated as low risk of bias due to time series design. |
| Exposure Assessment (Air Pollution) | Rated as probably high risk of bias because concentrations of SO_2_, NO_2_, PM_10_, and PM_2.5_ were measured twice a week at five air quality monitoring stations of the Karnataka State Pollution Control Board. |
| Exposure Assessment (Temperature) | Rated as probably low risk of bias because daily temperature data were obtained from a meteorological department in Bangalore. |
| Exposure Assessment (Pollen) | Pollen was not assessed in this study. |
| Detection of Outcome | Rated as low risk of bias because the outcome was classified using ICD codes for hospital visits related to wheezing, shortness of breath, difficulty breathing, and inhaler use or treatment of asthma. |
| Reporting | Rated as low risk of bias because all outcomes are reported. |
| Conflict of Interest | Rated as low risk of bias because the authors declare no competing interests. |
|  |  |
| Table S24. Lam et al 2016 | |
| Study Design | Rated as low risk of bias due to time series design. |
| Exposure Assessment (Air Pollution) | Rated as probably low risk of bias because average daily levels of RSP, SO_2_, NO_2_, and O_3_ were measured 11 general stations and 3 roadside stations of the Hong Kong Environmental Protection Department. |
| Exposure Assessment (Temperature) | Rated as low risk of bias because daily mean temperature data were obtained at a central monitoring station from the Hong Kong Observatory. |
| Exposure Assessment (Pollen) | Pollen was not assessed in this study. |
| Detection of Outcome | Rated as low risk of bias because the outcome was classified using ICD-9 codes for asthma hospitalizations. |
| Reporting | Rated as low risk of bias because all outcomes are reported. |
| Conflict of Interest | Rated as low risk of bias because the authors declare no competing interests. |
|  |  |
| Table S25. Lee et al 2018 | |
| Study Design | Rated as low risk of bias due to time-stratified, case-crossover design. |
| Exposure Assessment (Air Pollution) | Rated as probably low risk of bias because hourly concentrations of PM_2.5_, PM_10_, NO_2_, SO_2_, O_3_, and CO were measured at 27 monitoring sites (at least one centrally located site in each of Seoul’s 25 districts). |
| Exposure Assessment (Temperature) | Rated as low risk of bias because hourly data on ambient temperature was obtained from a weather monitoring station operated by the Korea Meteorological Administration. |
| Exposure Assessment (Pollen) | Pollen was not assessed in this study. |
| Detection of Outcome | Rated as low risk of bias because the outcome was classified using the ICD-10 code for ED visits for migraines. |
| Reporting | Rated as low risk of bias because all outcomes are reported. |
| Conflict of Interest | Rated as low risk of bias because the authors declare not conflict of interest. |
|  | |
| Table S26. Liu et al 2016 | |
| Study Design | Rated as low risk of bias due to time series design. |
| Exposure Assessment (Air Pollution) | Rated as probably high risk of bias because ozone concentrations are obtained from the NMMAPS database but other pollutants were not controlled for. |
| Exposure Assessment (Temperature) | Rated as low risk of bias because daily mean temperature data were obtained from the National Climatic Data Center through the NMMAPS database. |
| Exposure Assessment (Pollen) | Pollen was not assessed in this study. |
| Detection of Outcome | Rated as low risk of bias because the outcome was classified using ICD-9 and ICD-10 codes for non-accidental mortality. |
| Reporting | Rated as low risk of bias because all outcomes are reported. |
| Conflict of Interest | Rated as low risk of bias because the authors declare no conflict of interest. |
|  |  |
| Table S27. Lokys et al 2018 | |
| Study Design | Rated as probably low risk of bias due to meta-analysis design. |
| Exposure Assessment (Air Pollution) | Rated as probably high risk of bias because concentrations of NO_2_, SO_2_, O_3_, and PM_10_ were obtained from local air quality monitoring networks but it is unclear how many monitors were used or how data collection differed between districts. |
| Exposure Assessment (Temperature) | Rated as probably low risk hourly air temperature data were obtained from regional monitoring networks but no further details are given. |
| Exposure Assessment (Pollen) | Pollen was not assessed in this study. |
| Detection of Outcome | Rated as low risk of bias because the outcome was classified using ICD-10 codes for non-accidental hospital admissions that can be related to air pollution or thermal stress, including cardiovascular and respiratory diseases. |
| Reporting | Rated as low risk of bias because all outcomes are reported. |
| Conflict of Interest | Rated as probably low risk of bias because authors do not discuss conflict of interest. |
|  |  |
| Table S28. Luo et al 2017 | |
| Study Design | Rated as low risk of bias due to time series design. |
| Exposure Assessment (Air Pollution) | Rated as probably low risk of bias because concentrations of PM_10_, NO_2_, and SO_2_ were measured at the Chinese national air quality monitoring stations in each city (5-12 stations in each city). |
| Exposure Assessment (Temperature) | Rated as low risk of bias because minimum and maximum temperature data were obtained from the local meteorological administration in each city. |
| Exposure Assessment (Pollen) | Pollen was not assessed in this study. |
| Detection of Outcome | Rated as low risk of bias because the outcome was classified using ICD-10 codes for deaths due to cardiovascular disease. |
| Reporting | Rated as low risk of bias because all outcomes are reported. |
| Conflict of Interest | Rated as low risk of bias because the authors declare no competing financial interests. |
|  |  |
| Table S29. Makra et al 2015 | |
| Study Design | Rated as low risk of bias due to time series design. |
| Exposure Assessment (Air Pollution) | Rated as probably high risk of bias because concentrations of CO, NO, NO_2_, SO_2_, O_3_, and PM_10_ were measured at a single monitoring station located in the inner city area of Szeged. |
| Exposure Assessment (Temperature) | Rated as probably low risk of bias because daily minimum and maximum temperature data were measured at a monitoring station in the city. |
| Exposure Assessment (Pollen) | Rated as probably high risk of bias because pollen concentrations were measured using a Hirst-type volumetric trap but only at a single location. |
| Detection of Outcome | Rated as low risk of bias because the outcome is classified using ICD-10 codes for asthma ED visits. |
| Reporting | Rated as low risk of bias because all outcomes are reported. |
| Conflict of Interest | Rated as probably low risk of bias because the authors do not discuss conflict of interest. |
|  |  |
| Table S30. Matyasovszky et al 2011 | |
| Study Design | Rated as low risk of bias due to time series design. |
| Exposure Assessment (Air Pollution) | Rated as probably high risk of bias because concentrations of CO, NO, NO_2_, SO_2_, O_3_, and PM_10_ were measured at a single monitoring station located in the inner city area of Szeged. |
| Exposure Assessment (Temperature) | Rated as probably low risk of bias because mean temperature data were collected at a monitoring station in the city. |
| Exposure Assessment (Pollen) | Rated as probably high risk because the methods by which pollen concentrations were measured are unclear. |
| Detection of Outcome | Rated as probably low risk of bias because the outcome is hospital admissions for respiratory problems but it is unclear if ICD codes were used. |
| Reporting | Rated as low risk of bias because all outcomes are reported. |
| Conflict of Interest | Rated as probably low risk of bias because the authors do not discuss conflict of interest. |
|  |  |
| Table S31. Mazenq et al 2017 | |
| Study Design | Rated as low risk of bias due to time series design. |
| Exposure Assessment (Air Pollution) | Rated as probably low risk of bias because concentrations of PM_10_ and PM_2.5_ were measured by AIR PACA at between five to 17 monitoring stations in the region. |
| Exposure Assessment (Temperature) | Rated as low risk of bias because temperature data for each zip code were obtained from Météo-France using 151 monitoring stations. |
| Exposure Assessment (Pollen) | Rated as probably high risk because pollen data were obtained from two stationary spore traps in the region with some zip codes/communes being up to 100 km away from the nearest monitoring station. |
| Detection of Outcome | Rated as low risk of bias because the outcome is classified using ICD codes for asthma-related pediatric ED visits. |
| Reporting | Rated as low risk of bias because all outcomes are reported. |
| Conflict of Interest | Rated as low risk of bias because the authors declare no conflict of interest. |
|  |  |
| Table S32. Mirabelli et al 2016 | |
| Study Design | Rated as probably low risk of bias due to time series design but retrospective and self-reported |
| Exposure Assessment (Air Pollution) | Rated as probably high risk of bias because daily estimates of PM_2.5_ and O_3_ were generated for specific geographic locations using a Bayesian space-time Downscaler fusion model and EPA data. |
| Exposure Assessment (Temperature) | Rated probably high risk of bias because county-level estimates of ambient air temperature were generated using predictions from the North American Land Data Assimilation System Phase 2 model. |
| Exposure Assessment (Pollen) | Pollen is not assessed in this study. |
| Detection of Outcome | Rated as probably high risk of bias because the outcome is classified using self-reported asthma symptoms from survey respondents. |
| Reporting | Rated as low risk of bias because all outcomes are reported. |
| Conflict of Interest | Rated as low risk of bias because the authors declare no competing financial interests. |
|  |  |
| Table S33. Mireku et al 2009 | |
| Study Design | Rated as low risk of bias due to time series design. |
| Exposure Assessment (Air Pollution) | Rated as probably high risk of bias because county data for NO_2_, SO_2_, O_3_, CO, PM_10_ and PM_2.5_ were obtained from the EPA but it is unclear how many monitors were used. |
| Exposure Assessment (Temperature) | Rated as low risk of bias because minimum and maximum temperature data were measured at the Detroit City Airport weather station and obtained from the National Climatic Data Center. |
| Exposure Assessment (Pollen) | Rated as probably high risk of bias because aeroallergen data were obtained from one certified pollen station 17 miles from the study hospital. |
| Detection of Outcome | Rated as probably low risk of bias because the outcome is measured by patients presenting with a primary diagnosis of asthma exacerbation, but it is unclear if ICD codes were used. |
| Reporting | Rated as low risk of bias because all outcomes are reported. |
| Conflict of Interest | Rated as probably low risk of bias because the authors do not discuss conflict of interest. |
|  |  |
| Table S34. Moolgavkar et al 2003 | |
| Study Design | Rated as low risk of bias due to time series design. |
| Exposure Assessment (Air Pollution) | Rated as probably high risk of bias because daily concentrations of O_3_, SO_2_, NO_2_, and CO were obtained from the U.S. EPA and California EPA using all monitors in each county but the number of monitors is unclear. |
| Exposure Assessment (Temperature) | Rated as probably low risk of bias because temperature data were obtained from the airport monitoring stations in both counties but only seasonal. |
| Exposure Assessment (Pollen) | Pollen was not assessed in this study. |
| Detection of Outcome | Rated as low risk of bias because the outcome is classified by ICD-9 codes for mortality and specifically, cardiovascular deaths extracted from NCHS data. |
| Reporting | Rated as low risk of bias because all outcomes are reported. |
| Conflict of Interest | Rated as probably low risk of bias because the author does not discuss conflict of interest. |
|  |  |
| Table S35. Park et al 2011 | |
| Study Design | Rated as low risk of bias due to time series design. |
| Exposure Assessment (Air Pollution) | Rated as probably low risk of bias because hourly concentrations of PM_10_, NO_2_, SO_2_, CO and O_3_ were measured at 27 monitoring stations spanning the entire region of Seoul. |
| Exposure Assessment (Temperature) | Rated as low risk of bias because mean hourly temperature data were obtained from the National Meteorological Office using a monitoring station in central Seoul. |
| Exposure Assessment (Pollen) | Pollen was not assessed in this study. |
| Detection of Outcome | Rated as low risk of bias because the outcome is classified using ICD-10 codes for non-accidental mortality, cardiovascular mortality, and respiratory mortality. |
| Reporting | Rated as low risk of bias because all outcomes are reported. |
| Conflict of Interest | Rated as low risk of bias because the authors declare no conflict of interest. |
|  |  |
| Table S36. Pattenden et al 2010 | |
| Study Design | Rated as low risk of bias due to time series design. |
| Exposure Assessment (Air Pollution) | Rated as probably high risk of bias because concentrations of O_3_ and PM_10_  were measured by urban background air quality monitoring stations but the minimum for inclusion was having data available for at least 50% of days in the study period. |
| Exposure Assessment (Temperature) | Rated as probably low risk of bias because temperature data were obtained from meteorological stations of the British Atmosphere Data Centre. |
| Exposure Assessment (Pollen) | Pollen was not assessed in this study. |
| Detection of Outcome | Rated as low risk of bias because the outcome is classified using ICD-9 and ICD-10 codes for cardiovascular, respiratory, and other mortality. |
| Reporting | Rated as low risk of bias because all outcomes are reported. |
| Conflict of Interest | Rated as low risk of bias because the authors declare no conflict of interest. |
|  |  |
| Table S37. Peng et al 2013 | |
| Study Design | Rated as low risk of bias due to time series design. |
| Exposure Assessment (Air Pollution) | Rated as probably low risk of bias because daily measurements of O_3_ and PM_10_ were obtained from the air monitoring networks in each of the ~125 cities, which were selected per city-specific availability of air pollution data. |
| Exposure Assessment (Temperature) | Rated as probably low risk of bias because city-specific time-series data on daily temperature were obtained from pre-existing databases. |
| Exposure Assessment (Pollen) | Pollen was not assessed in this study. |
| Detection of Outcome | Rated as probably low risk of bias because the outcome is classified using ICD-9 codes for mortality and pre-existing databases. |
| Reporting | Rated as low risk of bias because all outcomes are reported. |
| Conflict of Interest | Rated as low risk of bias because the authors declare no conflict of interest. |
|  |  |
| Table S38. Qiu et al 2018 | |
| Study Design | Rated as low risk of bias due to time series design. |
| Exposure Assessment (Air Pollution) | Rated as probably low risk of bias because hourly concentrations of PM_2.5_, PM_10,_ SO_2_, NO_2_, CO and O_3_ were measured at six monitoring stations in five urban districts of Chengdu. |
| Exposure Assessment (Temperature) | Rated as probably low risk of bias because daily average temperature data was obtained from the Chengdu Meteorological Monitoring Database. |
| Exposure Assessment (Pollen) | Pollen was not assessed in this study. |
| Detection of Outcome | Rated as low risk of bias because the outcome is classified using ICD-10 codes for COPD hospital admissions. |
| Reporting | Rated as low risk of bias because all outcomes are reported. |
| Conflict of Interest | Rated as low risk of bias because the authors declare no conflict of interest. |
|  |  |
| Table S39. Rainham et al 2005 | |
| Study Design | Rated as low risk of bias due to time series design. |
| Exposure Assessment (Air Pollution) | Rated as probably low risk of bias because hourly concentrations of CO, NO_2_, SO_2_, O_3_, and PM_2.5_ were obtained from the Ontario Ministry of the Environment but it is unclear how many monitoring sites were used. |
| Exposure Assessment (Temperature) | Rated as probably low risk of bias because meteorological data were obtained from the Meteorological Service of Canada from a station at the Toronto Pearson International Airport. |
| Exposure Assessment (Pollen) | Pollen is not assessed in this study. |
| Detection of Outcome | Rated as low risk of bias because the outcome is classified using ICD-9 codes for cardiorespiratory and non-cardiorespiratory mortality and data from Statistics Canada. |
| Reporting | Rated as low risk of bias because all outcomes are reported. |
| Conflict of Interest | Rated as probably low risk of bias because the authors do not discuss conflict of interest. |
|  |  |
| Table S40. Ren et al 2008 | |
| Study Design | Rated as low risk of bias due to time series design. |
| Exposure Assessment (Air Pollution) | Rated as probably high risk of bias because only time series data for ozone were obtained from the EPA AIRS database and other pollutants were not controlled for. |
| Exposure Assessment (Temperature) | Rated as probably low risk of bias because daily maximum temperature data were obtained from the NMMAPS database for 95 communities. |
| Exposure Assessment (Pollen) | Pollen was not assessed in this study. |
| Detection of Outcome | Rated as low risk of bias because the outcome is classified using ICD-9 and ICD-10 codes for cardiovascular mortality. |
| Reporting | Rated as low risk of bias because all outcomes are reported. |
| Conflict of Interest | Rated as low risk of bias because the authors declare no competing interests. |
|  |  |
| Table S41. Ren et al 2009 | |
| Study Design | Rated as low risk of bias due to time series design. |
| Exposure Assessment (Air Pollution) | Rated as probably high risk of bias because only ozone concentrations were obtained from the EPA AIRS database and other pollutants were not controlled for. |
| Exposure Assessment (Temperature) | Rated as probably low risk of bias because daily maximum temperature data were obtained from the National Climatic Data Center for 95 communities. |
| Exposure Assessment (Pollen) | Pollen was not assessed in this study. |
| Detection of Outcome | Rated as low risk of bias because the outcome is classified using ICD-9 and ICD-10 codes for cardiovascular mortality. |
| Reporting | Rated as low risk of bias because all outcomes are reported. |
| Conflict of Interest | Rated as probably low risk of bias because the authors do not discuss conflict of interest. |
|  |  |
| Table S42. Ross et al 2002 | |
| Study Design | Rated as low risk of bias due to prospective cohort design. |
| Exposure Assessment (Air Pollution) | Rated as probably low risk of bias because concentrations of O_3_, PM_10_, and SO_2_ were obtained from the Illinois EPA and all participants lived within 8 km (5 mi) of a monitoring station. |
| Exposure Assessment (Temperature) | Rated as probably low risk of bias because meteorological data for the area was obtained from the National Climatic Data Center. |
| Exposure Assessment (Pollen) | Rated as probably low of bias because aeroallergens were measured at three sites in East Moline using Rotorod collection devices. |
| Detection of Outcome | Rated as probably high risk of bias because the outcome is measured using participant self-administered and self-reported peak expiratory flow rates. |
| Reporting | Rated as low risk of bias because all outcomes are reported. |
| Conflict of Interest | Rated as probably low risk of bias because the authors do not discuss conflict of interest. |
|  |  |
| Table S43. Scortichini et al 2018 | |
| Study Design | Rated as low risk of bias due to time series design. |
| Exposure Assessment (Air Pollution) | Rated as probably low risk of bias because concentrations of PM_10_ and O_3_ were obtained from the regional air quality monitoring network of each city and monitors had to meet the requirements of EPIAIR2 protocol. |
| Exposure Assessment (Temperature) | Rated as probably low risk of bias because temperature data was obtained from the airport station located closest to each city. |
| Exposure Assessment (Pollen) | Pollen was not assessed in this study. |
| Detection of Outcome | Rated as low risk of bias because the outcome was classified using ICD-9 and ICD-10 codes for non-accidental mortality. |
| Reporting | Rated as low risk of bias because all outcomes are reported. |
| Conflict of Interest | Rated as low risk of bias because the authors declare no conflict of interest. |
|  |  |
|  |  |
| Table S44. Shaposhnikov et al 2014 | |
| Study Design | Rated as low risk of bias due to time series design. |
| Exposure Assessment (Air Pollution) | Rated as probably low risk of bias because PM_10_ and O_3_ measurements were taken at between six and 13 monitoring stations in Moscow using a standardized method over the course of the study period. |
| Exposure Assessment (Temperature) | Rated as low risk of bias because average daily temperature data were obtained from the Meteorological Observatory of the Moscow State University. |
| Exposure Assessment (Pollen) | Pollen was not assessed in this study. |
| Detection of Outcome | Rated as low risk of bias because the outcome is classified using ICD-10 codes for mortality. |
| Reporting | Rated as low risk of bias because all outcomes are reported. |
| Conflict of Interest | Rated as probably low risk of bias because the authors do not discuss conflict of interest. |
|  |  |
| Table S45. Silverberg et al 2015 | |
| Study Design | Rated as low risk of bias due to time series design. |
| Exposure Assessment (Air Pollution) | Air pollution is not assessed in this study, only stratospheric ozone levels are included as a marker for UV exposure. |
| Exposure Assessment (Temperature) | Rated as probably low risk of bias because statewide temperature data were obtained from the NOAA, National Climate Data Center. |
| Exposure Assessment (Pollen) | Rated as high risk of bias because pollen counts were obtained from the National Allergy Bureau but were only available for 27 sites across 21 states and districts. |
| Detection of Outcome | Rated as probably low risk of bias because the outcome is classified by asking respondents if their child has been diagnosed with hay fever or respiratory allergy by a doctor or health professional in the last 12 months. |
| Reporting | Rated as low risk of bias because all outcomes are reported. |
| Conflict of Interest | Rated as low risk of bias because the authors declare no conflict of interest. |
|  |  |
| Table S46. Stafoggia et al 2008 | |
| Study Design | Rated as low risk of bias due to case-crossover design. |
| Exposure Assessment (Air Pollution) | Rated as probably high risk of bias because hourly PM_10_ data were obtained from city monitors in residential areas but other pollutants were not measured or controlled for. |
| Exposure Assessment (Temperature) | Rated as probably low risk of bias because air temperature data were obtained from the Italian Air Force Meteorological Service. |
| Exposure Assessment (Pollen) | Pollen was not assessed in this study. |
| Detection of Outcome | Rated as low risk of bias because the outcome is classified using ICD-9 codes for deaths due to cardiovascular diseases, respiratory diseases, and other natural diseases. |
| Reporting | Rated as low risk of bias because all outcomes are reported. |
| Conflict of Interest | Rated as low risk of bias because the authors declare no conflict of interest. |
|  |  |
| Table S47. Stieb et al 2000 | |
| Study Design | Rated as low risk of bias due to time series design. |
| Exposure Assessment (Air Pollution) | Rated as high risk of bias because CO, H_2_S, NO_2_, O_3_, SO_2_, PM_2.5_, PM_10_, TRS, and COH were measured but only one monitoring site was in operation for the duration of the entire study period. |
| Exposure Assessment (Temperature) | Rated as low risk of bias because temperature data were collected at the Saint John airport and obtained from Environment Canada. |
| Exposure Assessment (Pollen) | Rated as probably high risk of bias because aeroallergen data was collected using rotation impaction sampling equipment but only at two sites. |
| Detection of Outcome | Rated as probably low risk of bias because the outcome is classified by a manual review of cardiorespiratory ED visits by a health records coder. |
| Reporting | Rated as low risk of bias because all outcomes are reported. |
| Conflict of Interest | Rated as probably low risk of bias because the authors do not discuss conflict of interest. |
|  |  |
| Table S48. Vanos et al 2015 | |
| Study Design | Rated as low risk of bias due to time series design. |
| Exposure Assessment (Air Pollution) | Rated as probably low risk of bias because concentrations of O_3_, NO_2_, PM_2.5_ and SO_2_ were obtained from the National Air Pollution Surveillance Network database but it is unclear how many monitors were used. |
| Exposure Assessment (Temperature) | Rated as low risk of bias because temperature data were measured at first-order airport weather stations maintained by the Meteorological Service of Canada for each of the 12 cities. |
| Exposure Assessment (Pollen) | Pollen was not assessed in this study. |
| Detection of Outcome | Rated as low risk of bias because the outcome is classified using ICD-9 and ICD-10 codes for non-accidental mortality and data from Statistics Canada. |
| Reporting | Rated as low risk of bias because all outcomes are reported. |
| Conflict of Interest | Rated as probably low risk of bias because the authors do not discuss conflict of interest. |
|  | |
| Table S49. Wilson et al 2014 | |
| Study Design | Rated as low risk of bias due to time series design. |
| Exposure Assessment (Air Pollution) | Rated as probably high risk of bias because only ozone concentrations are obtained through the NMMAPS database and other pollutants are not controlled for. |
| Exposure Assessment (Temperature) | Rated as low risk of bias because temperature data were obtained from the NMMAPS database for the 95 cities. |
| Exposure Assessment (Pollen) | Pollen was not assessed in this study. |
| Detection of Outcome | Rated as probably low risk of bias because mortality data is obtained from the NMMAPS database but further details are unclear. |
| Reporting | Rated as low risk of bias because all outcomes are reported. |
| Conflict of Interest | Rated as probably low risk of bias because the authors do not discuss conflict of interest. |
|  |  |
| Table S50. Winquist et al 2014 | |
| Study Design | Rated as low risk of bias due to time series design. |
| Exposure Assessment (Air Pollution) | Rated as probably low risk of bias because daily concentrations of CO, NO_2_, SO_2_, O_3_, PM_2.5_ and its components were obtained from several ambient monitoring networks in Atlanta. |
| Exposure Assessment (Temperature) | Rated as probably high risk of bias because temperature is controlled for, but it is unclear how the meteorological data were obtained and analysis is only seasonal. |
| Exposure Assessment (Pollen) | Pollen was not assessed in this study. |
| Detection of Outcome | Rated as low risk of bias because the outcome is classified using ICD-9 codes for pediatric asthma ED visits. |
| Reporting | Rated as low risk of bias because all outcomes are reported. |
| Conflict of Interest | Rated as probably low risk of bias because the authors do not discuss conflict of interest. |
|  |  |
| Table S51. Witonsky et al 2018 | |
| Study Design | Rated as low risk of bias due to time series design. |
| Exposure Assessment (Air Pollution) | Rated as probably high risk of bias because concentrations of NO_x_, O_3_, PM_2.5_, and SO_2_ were each only measured at one station in the Bronx. |
| Exposure Assessment (Temperature) | Rated as low risk of bias because temperature data were obtained from the National Climatic Data Center’s Bronx station. |
| Exposure Assessment (Pollen) | Rated as probably high risk of bias because daily pollen counts were obtained from a single monitoring station 21 miles north of the Bronx using a Burkhard sampler. |
| Detection of Outcome | Rated as low risk of bias because the outcome is classified using ICD-9 codes for asthma ED visits and asthma hospitalizations. |
| Reporting | Rated as low risk of bias because all outcomes are reported. |
| Conflict of Interest | Rated as low risk of bias because the authors declare no conflict of interest. |
|  |  |
| Table S52. Zhang et al 2006 | |
| Study Design | Rated as low risk of bias due to time series design. |
| Exposure Assessment (Air Pollution) | Rated as probably low risk of bias because concentrations of O_3_, PM_10_, SO_2_, and NO_2_ were collected by the Shanghai Environmental Monitoring Center at six urban stations. |
| Exposure Assessment (Temperature) | Rated as low risk of bias because temperature data were collected by the Shanghai Meteorological Bureau. |
| Exposure Assessment (Pollen) | Pollen was not assessed in this study. |
| Detection of Outcome | Rated as low risk of bias because the outcome is classified using ICD-9 and ICD-10 codes for mortality. |
| Reporting | Rated as low risk of bias because all outcomes are reported. |
| Conflict of Interest | Rated as low risk of bias because the authors declare no competing interests. |

| Table S53. Cheng et al 2015 | |
| --- | --- |
| Study Design | Rated as low risk of bias due to time series design. |
| Exposure Assessment (Air Pollution) | Rated as probably low risk of bias because concentrations of O_3_, PM_10_, SO_2_, and NO_2_ were obtained from the Shanghai Environmental Monitoring Center database and were measured at six urban stations. |
| Exposure Assessment (Temperature) | Rated as low risk of bias because minimum, maximum, and mean temperature values were obtained from the Shanghai Meteorological Bureau. |
| Exposure Assessment (Pollen) | Pollen was not assessed in this study. |
| Detection of Outcome | Rated as low risk of bias because the outcome is classified using ICD-9 and ICD-10 codes for nonaccidental deaths, as well as deaths due to cardiovascular and respiratory diseases. |
| Reporting | Rated as low risk of bias because all outcomes are reported. |
| Conflict of Interest | Rated as low risk of bias because the authors declare no competing interests. |

| Table S54. Li et al 2011 | |
| --- | --- |
| Study Design | Rated as low risk of bias due to time series design. |
| Exposure Assessment (Air Pollution) | Rated as probably low risk of bias because concentrations of PM_10_, NO_2_, and SO_2_ were measured at 12 monitoring stations in both urban and suburban areas of Tianjin. |
| Exposure Assessment (Temperature) | Rated as probably low risk of bias because temperature data were obtained from the National Meteorological Information Center. |
| Exposure Assessment (Pollen) | Pollen was not assessed in this study. |
| Detection of Outcome | Rated as low risk of bias because the outcome is classified using ICD-10 codes for nonaccidental, cardiovascular, respiratory, cardiopulmonary, stroke, and IHD mortalities. |
| Reporting | Rated as low risk of bias because all outcomes are reported. |
| Conflict of Interest | Rated as probably low risk of bias because the authors do not discuss conflict of interest. |

| Table S55. Li et al 2015 | |
| --- | --- |
| Study Design | Rated as low risk of bias due to time series design. |
| Exposure Assessment (Air Pollution) | Rated as probably high risk of bias because concentrations of PM_10_ are measured at seven monitoring stations but no other pollutants are controlled for. |
| Exposure Assessment (Temperature) | Rated as low risk of bias because daily mean temperature data were obtained from the Guangzhou Weather Station. |
| Exposure Assessment (Pollen) | Pollen was not assessed in this study. |
| Detection of Outcome | Rated as low risk of bias because the outcome is classified by ICD-10 codes for nonaccidental, cardiovascular, and respiratory deaths. |
| Reporting | Rated as low risk of bias because all outcomes are reported. |
| Conflict of Interest | Rated as low risk of bias because the authors declare no conflict of interest. |

| Table S56. Meng et al 2012 | |
| --- | --- |
| Study Design | Rated as low risk of bias due to time series design. |
| Exposure Assessment (Air Pollution) | Rated as probably low risk of bias because PM_10_ data for the eight cities is obtained from the National Air Pollution System but other pollutants are not controlled for. |
| Exposure Assessment (Temperature) | Rated as probably low risk of bias because temperature data were obtained from the National Meteorological Information Center. |
| Exposure Assessment (Pollen) | Pollen was not assessed in this study. |
| Detection of Outcome | Rated as low risk of bias because the outcome is classified by ICD-10 codes for nonaccidental, cardiovascular, and respiratory deaths. |
| Reporting | Rated as low risk of bias because all outcomes are reported. |
| Conflict of Interest | Rated as low risk of bias because the authors declare no competing interests. |

| Table S57. Sun et al 2015 | |
| --- | --- |
| Study Design | Rated as low risk of bias due to time series design. |
| Exposure Assessment (Air Pollution) | Rated as probably low risk of bias because concentrations of PM_2.5_, PM_10_, SO_2_, NO_2_, and O_3_ were measured by the Environmental Protection Department of Hong Kong at ten monitoring stations. |
| Exposure Assessment (Temperature) | Rated as low risk of bias because temperature data were obtained from the Hong Kong Observatory. |
| Exposure Assessment (Pollen) | Pollen was not assessed in this study. |
| Detection of Outcome | Rated as low risk of bias because the outcome is classified by ICD-9 and ICD-10 codes for nonaccidental, cardiovascular, and respiratory deaths. |
| Reporting | Rated as low risk of bias because all outcomes are reported. |
| Conflict of Interest | Rated as low risk of bias because the authors report no competing interests. |
